# Bimetal‐Phenolic Framework to Combat Bacterial Infections via Synergistic Biofilm Dispersal, Bacterial Killing and Immune Modulation

**DOI:** 10.1002/advs.202513863

**Published:** 2025-09-17

**Authors:** Yaran Wang, Fan Wu, Lei Hua, Chang Gao, Siran Wang, Yong Liu, Yijin Ren, Linqi Shi, Henny C. van der Mei, Yuanfeng Li

**Affiliations:** ^1^ Consortium for Infection and Innovation (CII) Translational Medicine Laboratory the First Affiliated Hospital of Wenzhou Medical University Wenzhou Zhejiang 325035 China; ^2^ State Key Laboratory of Medicinal Chemical Biology Key Laboratory of Functional Polymer Materials Ministry of Education Institute of Polymer Chemistry College of Chemistry Nankai University Tianjin 300071 China; ^3^ Department of Biomaterials & Biomedical Technology University of Groningen and University Medical Center Groningen Groningen 9713 AV Netherlands; ^4^ Department of Orthodontics University of Groningen and University Medical Center Groningen Groningen 9700 RB Netherlands; ^5^ Wenzhou Institute University of Chinese Academy of Sciences Wenzhou Zhejiang 325001 China

**Keywords:** anti‐inflammatory, biofilm degradation, catalytic activity, lung infections, multifunctional nanomaterials

## Abstract

The presence of drug resistance and extracellular matrix protection in biofilms makes it increasingly difficult to control bacterial infections using antibiotics. Therefore, there is an urgent need to develop new non‐antibiotic approaches to eradicate drug‐resistant bacterial infections. Here, a bimetal‐phenolic framework (Que‐Fe‐CeMPF) is constructed by direct self‐assembly of coordinated Fe^3+^ and Ce^4+^ ions with the polyphenol quercetin (Que). Que‐Fe‐CeMPF enhanced hydroxyl radical (·OH) generation, particularly in an acidic environment and presence of H_2_O_2,_ compared with single metal‐phenolic frameworks (Que‐FeMPF and Que‐CeMPF). · OH damaged bacterial cell walls, resulting in intracellular protein loss and bacterial cell death. Additionally, Que‐Fe‐CeMPF effectively dispersed biofilms by degrading matrix eDNA, allowing easier ·OH penetration, resulting in higher killing efficiency compared to Que‐FeMPF and Que‐CeMPF. Que‐Fe‐CeMPF stimulated macrophages to adopt an M2‐like phenotype, suppressing excessive immune activation and promoting tissue repair at the infection site. As a combined effect of bacterial killing, biofilm degradation, and immune‐modulation, the infectious pneumonia caused by *Pseudomonas aeruginosa* in mice is more effectively eradicated by Que‐Fe‐CeMPF than by free quercetin or the antibiotic ciprofloxacin. Moreover, Que‐Fe‐CeMPF is less prone to resistance development in pathogens compared to ciprofloxacin. Thus, Que‐Fe‐CeMPF is a promising non‐antibiotic antimicrobial agent with multimodal activity for controlling drug‐resistant bacterial infections.

## Graphical abstract

A bimetal‐phenolic framework (Que‐Fe‐CeMPF) is developed by direct self‐assembly through the coordination of Fe^3+^ and Ce^4+^ with quercetin. Que‐Fe‐CeMPF exhibits multimodal activity, including cleaving matrix eDNA within biofilm, generating hydroxyl radicals via catalytic H_2_O_2_ to kill bacteria, and alleviating inflammation by stimulating macrophage polarization to adopt a M2‐like phenotype at the infection site.

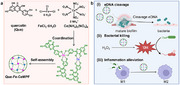

## 1 Introduction

The growing crisis of antibiotic resistance is largely driven by the overuse and misuse of antibiotics, combined with a lack of innovation in new drug development by the pharmaceutical industry.^[^ ^1^ ^]^ Since the first report of penicillin‐resistant *Staphylococcus* in 1948, drug‐resistant bacterial infections have become a major challenge and burden on global healthcare systems.^[^ ^2^, ^3^ ^]^ Conventional antibiotics are becoming increasingly ineffective, particularly against infections caused by multidrug‐resistant pathogens.^[^ ^4^ ^]^ To address this issue, numerous non‐antibiotic antibacterial strategies have been developed in recent years, utilizing antibacterial mechanisms distinct from those of conventional antibiotics.^[^ ^5^, ^6^ ^]^ However, bacteria in biofilms are protected from environmental attacks, which not only prevents antibacterial agents from penetrating the biofilm and effectively killing the bacteria, but also impairs clearance by the host immune system.^[^ ^7^ ^]^ Consequently, eliminating biofilm is significantly more challenging than targeting planktonic bacteria. Therefore, the development of new therapeutics with multimodal activity, such as bacterial killing, biofilm disruption, and immune regulation, remains a critical challenge.

Reactive oxygen species (ROS) are a group of highly reactive and short‐lived molecules with strong oxidative properties, including hydroxyl radicals (·OH) and singlet oxygen (^1^O_2_).^[^ ^8^ ^]^ These compounds can disrupt bacterial cell membranes, damage DNA, and modulate inflammation. ROS have been regarded as an effective antibacterial strategy to combat drug‐resistant bacteria.^[^ ^9^, ^10^ ^]^ Chemodynamic therapy can convert overexpressed hydrogen peroxide (H_2_O_2_) in the acidic microenvironment of infection sites into ·OH via Fenton or Fenton‐like reactions, enabling selective bacterial killing at the infection site. A key advantage of this therapy is the selective generation of ·OH at acidic infection sites for effectively eliminating pathogens while maintaining biosafety for surrounding healthy tissues. However, due to the presence of extracellular polymeric substances (EPS) in biofilms and the short lifespan of ·OH, penetration into the biofilm is limited.^[^ ^11^, ^12^ ^]^ Extracellular DNA (eDNA), a key component of EPS, plays an essential role in maintaining biofilm structure and has emerged as a strategic target for biofilm disruption. Once eDNA is degraded, biofilms begin to disintegrate, facilitating bacterial elimination. Although deoxyribonuclease (DNase) can hydrolyze eDNA in biofilms, its catalytic activity is affected by pH, the presence of Ca^2+^, and temperature.^[^ ^13^, ^14^ ^]^ Additionally, isolation and production of natural enzymes are complex and costly, limiting their practical application. In contrast, nanozymes offer several advantages, including high catalytic stability, ease of surface modification, and lower production costs.^[^ ^15^ ^]^ Therefore, nanozymes with multimodal activities, capable of hydrolyzing eDNA and killing bacteria within biofilms, may offer an effective solution for addressing drug‐resistant infections.^[^ ^16^, ^17^ ^]^

Metal‐organic frameworks, as promising porous materials with a high surface area and high catalytic activity, have emerged as a new generation of antibacterial agents with potential applications.^[^ ^18^ ^]^ Polyphenols, a class of polyhydroxy phenolic compounds, have potent antioxidant and anti‐inflammatory properties, and can protect tissue cells from oxidative damage induced by free radicals. They also modulate the immune response by promoting the production of anti‐inflammatory cytokines while suppressing proinflammatory cytokines, making them valuable in the prevention of inflammation‐related diseases.^[^ ^19^ ^]^ Polyphenols, as ligands, can easily coordinate with metal ions to form metal‐phenolic frameworks (MPFs).^[^ ^20^ ^]^ Metal ions play an essential catalytic role in various reactions. For example, in the Fenton reaction, Fe^2+^ catalyzes the redox reaction of H_2_O_2,_ accelerating the ·OH generation, particularly in acidic environments.^[^ ^21^ ^]^ Ce^4+^ ions can directly interact with phosphorylated, double‐bound oxygen atoms to weaken the phosphodiester bond in eDNA.^[^ ^22^ ^]^ Therefore, integrating these components into a single MPF system holds great promise for simultaneously dispersing biofilms, eradicating drug‐resistant bacteria, and modulating the immune response.

Here, a bimetal‐phenolic framework (Que‐Fe‐CeMPF) was synthesized by coordinating Fe^3+^ and Ce^4+^ ions with quercetin (Que). After its characterization, the catalytic activity of the Que‐Fe‐CeMPF was determined. Its bacterial killing and biofilm dispersal capabilities were evaluated in vitro against *Staphylococcus aureus* Xen36 and *Pseudomonas aeruginosa* Xen41. The influence of Que‐Fe‐CeMPF on the gene expression of *P. aeruginosa* ATCC27585 was determined through transcriptome analysis based on RNA sequencing. Finally, the in vivo anti‐infection capacity of Que‐Fe‐CeMPF was investigated by treating *P. aeruginosa* Xen41‐induced pneumonia in mice.

## 2 Results

### 2.1 Characterization of Que‐Fe‐CeMPF

**Figure 1:**
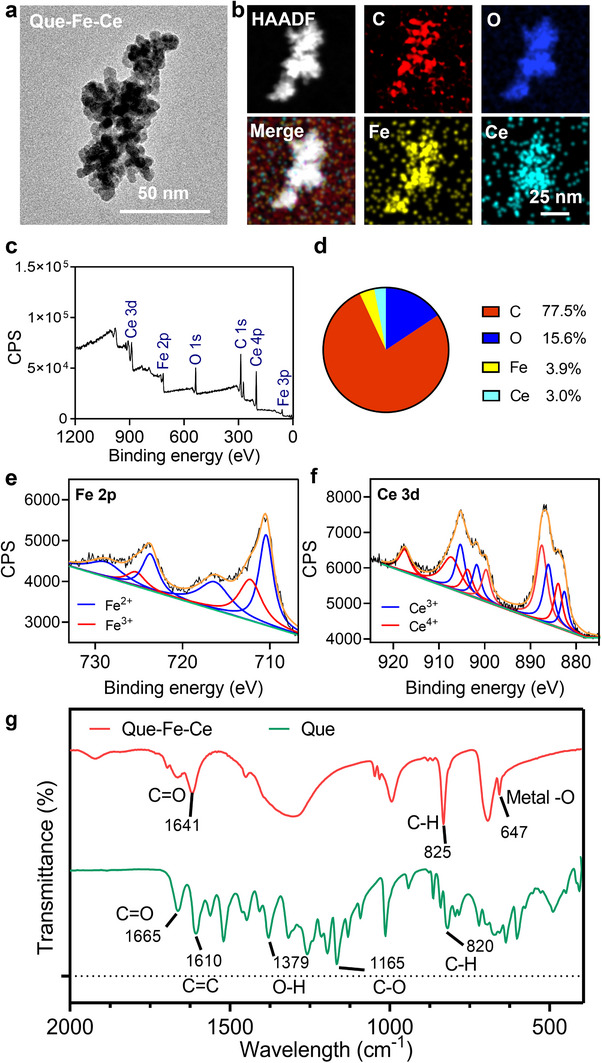
Morphology and chemical characterization of Que‐Fe‐CeMPF. a) TEM image of Que‐Fe‐CeMPF. b) High‐angle annular dark‐field (HAADF) and the element distribution of C, O, Fe, and Ce. c) Wide scan XPS spectrum and d) element composition determined from the XPS wide scan spectrum. e) XPS high resolution spectra of Fe 2p and f) same as panel e, now for Ce 3d. g) FT‐IR absorption spectra of Que‐Fe‐CeMPF and Que.

The MPFs of Que‐Fe, Que‐Ce, and Que‐Fe‐Ce, each containing different metal ions, were prepared by a one‐step direct assembly process in sodium carbonate buffer. Que‐Fe, Que‐Ce, and Que‐Fe‐Ce exhibited morphologies consisting of many small spherical structures interconnected to form an irregular network, see SEM and TEM images (**Figure** **1**a; Figure S1a, Supporting Information). The zeta potentials of all three MPFs were ≈‐30 mV (Figure S1b, Supporting Information). The elemental composition of Que‐Fe‐CeMPF was mapped using high‐angle annular dark‐field scanning TEM (HAADF‐STEM), which showed a uniform distribution of carbon, oxygen, iron, and cerium (Figure 1b). Quantitative analysis of the wide‐scan XPS spectrum indicated the elemental composition of Que‐Fe‐CeMPF to be ≈77% carbon, 16% oxygen, 4% iron, and 3% Ce (Figure 1c,d). The high‐resolution Fe 2p narrow scan (Figure 1e) showed characteristic peaks at 710.4 eV (Fe 2p_3/2_) and 723.7 eV (Fe 2p_1/2_), corresponding to Fe^2+^. The peaks observed at 712.2 eV (Fe 2p_3/2_) and 725.3 eV (Fe 2p_1/2_) are attributed to Fe^3+^. Additionally, satellite peaks at 716.3 and 728.8 eV further confirmed the presence of Fe^2+^.^[^ ^23^ ^]^ The Ce 3d peak in the narrow scan (Figure 1f) was decomposed into multiple peaks at 882.5 eV (Ce 3d_5/2_), 886.0 eV (Ce 3d_5/2_), 901.9 eV (3d_3/2_), and 905.5 eV (3d_3/2_), corresponding to Ce^3+^. The peaks observed at 883.9 eV (Ce 3d_5/2_), 887.5 eV (Ce 3d_5/2_), 890.0 eV (Ce 3d_5/2_), 904.0 eV (Ce 3d_3/2_), 907.0 eV (Ce 3d_3/2_), and 917.8 eV (Ce 3d_3/2_) are attributed to Ce^4+^.^[^ ^24^ ^]^ The FT‐IR spectrum of Que exhibited peaks at 1665 and 1165 cm^−1^, corresponding to the stretching vibrations of C ═ O and C─O. A peak at 1610 cm^−1^ was attributed to the aromatic stretching vibrations, consistent with the structure of Que (Figure 1g).^[^ ^25^ ^]^ Due to the interaction between metal ions (Fe and Ce) and Que, the C ═ O stretching vibration shifted from 1665 to 1641 cm^−1^, while the bending vibration of aromatic C─H shifted from 820 to 825 cm^−1^. Additionally, a peak at 647 cm^−1^, attributed to the stretching vibrations of metal‐O, confirmed successful metal coordination in Que‐Fe‐CeMPF.^[^ ^26^, ^27^ ^]^

### 2.2 Catalytic Activity of Que‐Fe‐CeMPF

**Figure 2:**
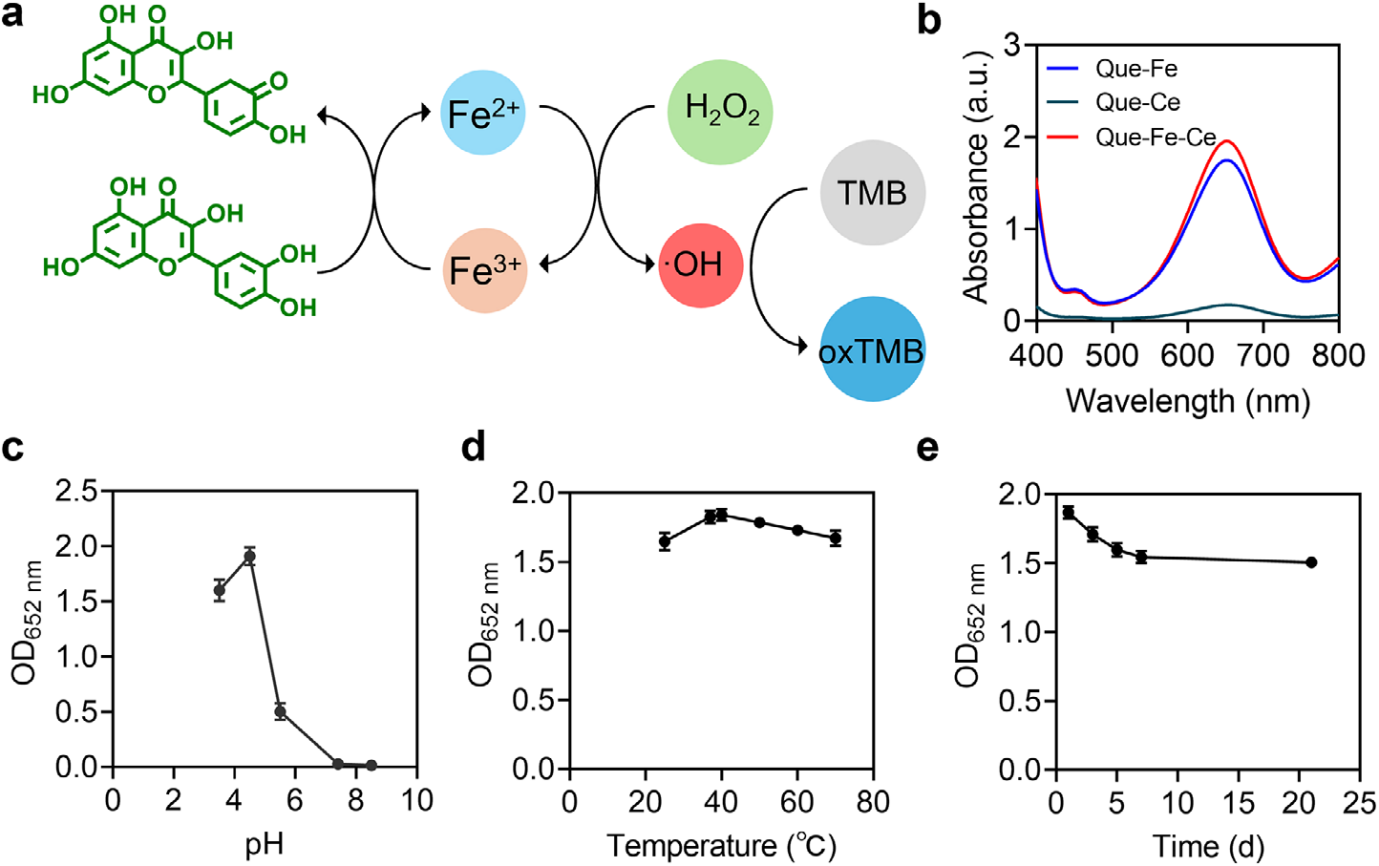
Catalytic activity of Que‐Fe‐CeMPF. a) Schematic illustration of TMB oxidation by Que‐Fe‐CeMPF. b) UV–vis absorption spectra of TMB oxidation of 50 µm Que‐Fe, Que‐Ce, and Que‐Fe‐Ce MPFs in the presence of H_2_O_2_ (100 µm) for 10 min at pH 4.5. c) UV–vis absorbance at 652 nm of TMB oxidation by 50 µm Que‐Fe‐CeMPF in the presence of H_2_O_2_ (100 µm) at different pHs (3.5, 4.5, 5.5, 7.4, and 8.5) for 10 min. d) Oxidation reaction performed at pH 4.5 under different temperatures (25, 37, 40, 50, 60, and 70 °C) and e) after different storage durations (1, 3, 5, 7, 21 days) of the MPF in a desiccator with SiO_2_. All experiments were done in triplicate.

The catalytic activity of Que‐Fe‐CeMPF was evaluated using the oxidation of 3,3′,5,5′‐tetra‐methyl benzidine (TMB), as illustrated in **Figure** **2**a. In the presence of non‐radical H_2_O_2_, Que‐Fe‐CeMPF exhibited the highest catalytic activity under acidic conditions (pH 4.5), indicated by the characteristic UV–vis absorption peak at 652 nm. Comparative analysis demonstrated that the bimetallic Que‐Fe‐CeMPF had a slightly higher catalytic activity compared to single‐metal Que‐FeMPF (Figure 2b). In contrast, catalytic activity was negligible at physiological pH (Figure 2c) and nearly absent without H_2_O_2_ (Figure S2a, Supporting Information). Moreover, the catalytic activity of free metal ions (Fe^3+^, Ce^4+^, Fe^3+^ + Ce^4+^), free Que, and single mixtures of Que with metal ions was lower than Que‐Fe‐CeMPF (Figure S2b,c, Supporting Information). Also, the stability of the catalytic activity of Que‐Fe‐CeMPF was measured under various temperatures and after long‐term storage in a desiccator with SiO_2_ at room temperature. As shown in Figure 2d, temperature had a minimal effect on the catalytic activity, and the MPF maintained its catalytic efficiency even after extended storage (Figure 2e). From the Lineweaver–Burk plot (Figure S3, Supporting Information), the corresponding kinetic parameters, including the maximum initial velocity (*V_max_*) and the Michaelis‐Menten constant (*K_m_*), were obtained. The steady‐state kinetics for the TMB oxidation reaction of Que‐Fe‐Ce yielded a *V_max_* of 442.3 nM s^−1^ and a *K_m_* of 10.70 mm, showing a higher reaction activity compared to Fe_3_O_4_ (*V_max_* of 97.8 nM s^−1^ and *K_m_* of 154 mm).^[^ ^28^ ^]^

### 2.3 Antimicrobial Activity of Que‐Fe‐CeMPF In Vitro

**Figure 3:**
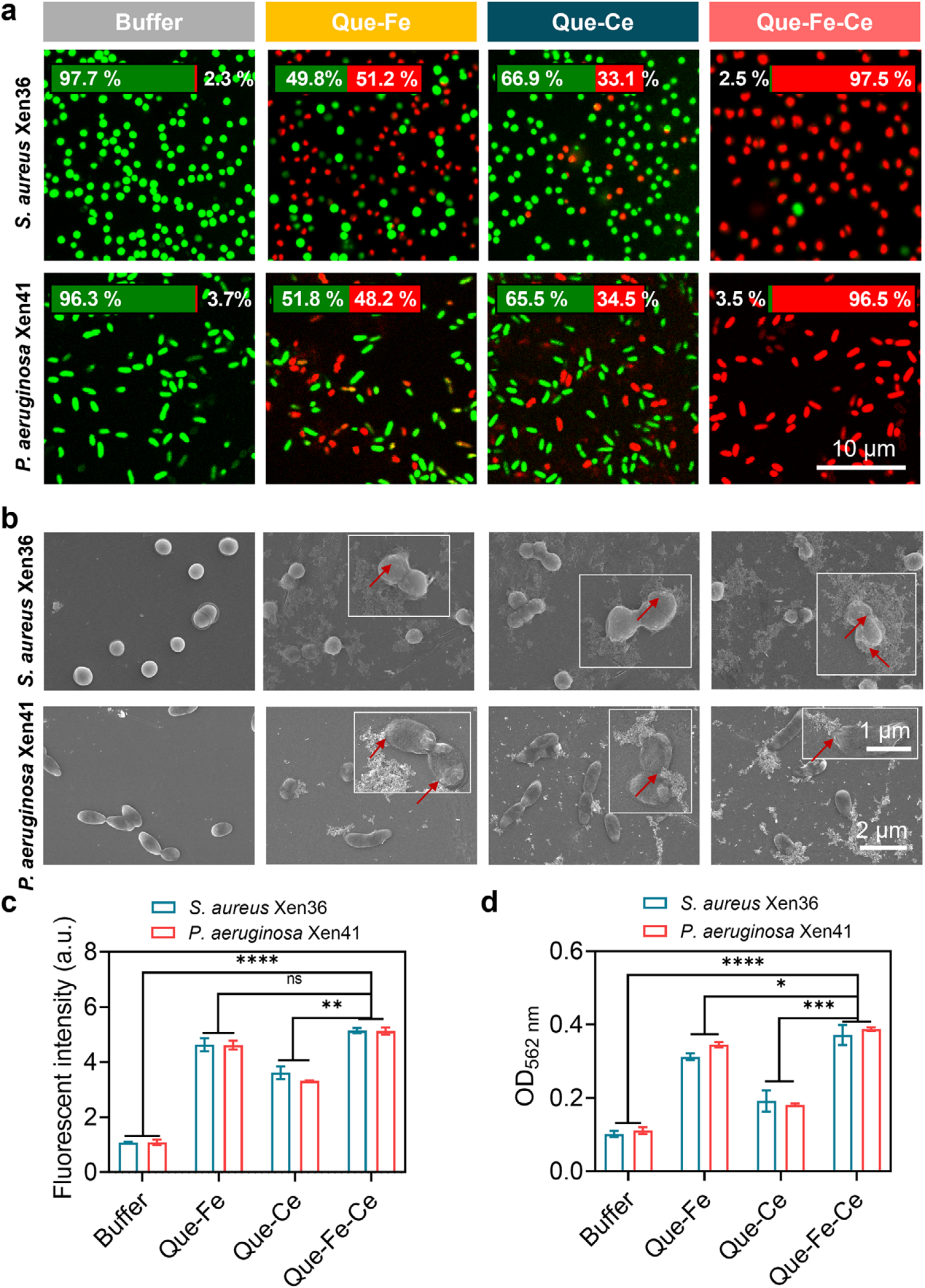
Antimicrobial properties of Que‐FeMPF, Que‐CeMPF, and Que‐Fe‐CeMPF in sodium acetate buffer at pH 4.5. a) CLSM images of planktonic *S. aureus* Xen36 and *P. aeruginosa* Xen41 exposed to 50 µm MPFs at pH 4.5 for 3 h in the presence of H_2_O_2_ (100 µm), showing live (green) and membrane‐damaged (red) bacteria. Insets quantify fluorescence intensity ratios. b) Same as panel a, now SEM images of *S. aureus* Xen36 and *P. aeruginosa* Xen41. Insets at higher magnifications with red arrows pointing to the damaged cell wall. c) ROS production by *S. aureus* Xen36 and *P. aeruginosa* Xen41 after exposure to the MPFs under the same conditions as panel a by using a fluorescent ROS probe (DCFH‐DA). d) Release of intracellular proteins from *S. aureus* Xen36 and *P. aeruginosa* Xen41 after exposure to the MPFs under the same conditions as panel a. The intracellular proteins were measured using a bicinchoninic acid (BCA) test kit. *p < 0.05, **p < 0.01, ***p < 0.001, and ****p < 0.0001 indicate statistical significance (one‐way ANOVA) over the differences indicated by the spanning bars.

To investigate the antibacterial efficacy of Que‐Fe‐CeMPF in vitro, planktonic *S. aureus* and *P. aeruginosa* were treated with varying concentrations of Que‐Fe‐CeMPF in the presence or absence of H_2_O_2_ at pH 4.5. A concentration‐dependent decrease in CFU was observed, with significantly higher bacterial killing in the presence of H_2_O_2_ for both bacterial species (Figure S4, Supporting Information). *S. aureus* Xen36 and *P. aeruginosa* Xen41 were also treated with different MPFs in the absence and presence of H_2_O_2_ at pH 7.4 or 4.5 (Figure S5, Supporting Information). At pH 7.4, no significant reduction in CFUs was observed regardless of H_2_O_2_ concentration. In contrast, at pH 4.5, especially in the presence of H_2_O_2_, all MPFs showed a significant reduction in CFUs, due to ·OH generation. Que alone showed no significant antibacterial effect at a concentration of 50 µm, with or without H_2_O_2_. Exposure of planktonic bacteria to Que‐Fe‐CeMPF in the presence of H_2_O_2_ at pH 4.5 led to 97% killing of both bacterial strains (**Figure** **3**a). Que‐Ce‐Fe MPFs outperformed both Que‐Fe and Que‐Ce, demonstrating a synergistic effect between Fe and Ce ions. Scanning electron microscopy revealed that MPF‐treated bacteria exhibited cell wall shrinkage and damage, and appeared to be entrapped by MPFs networks (Figure 3b). This suggests that MPFs physically interact with the bacterial surface, acting like ‘a fishing net’ that captures and subsequently damages the cell wall through ·OH generation (Figure 3c). The cell wall damage was confirmed by the release of intracellular proteins (Figure 3d). Due to the non‐specific antibacterial mechanism of ·OH, similar efficacy was observed against both *S. aureus* and *P. aeruginosa*.

### 2.4 Transcriptome Analysis

**Figure 4:**
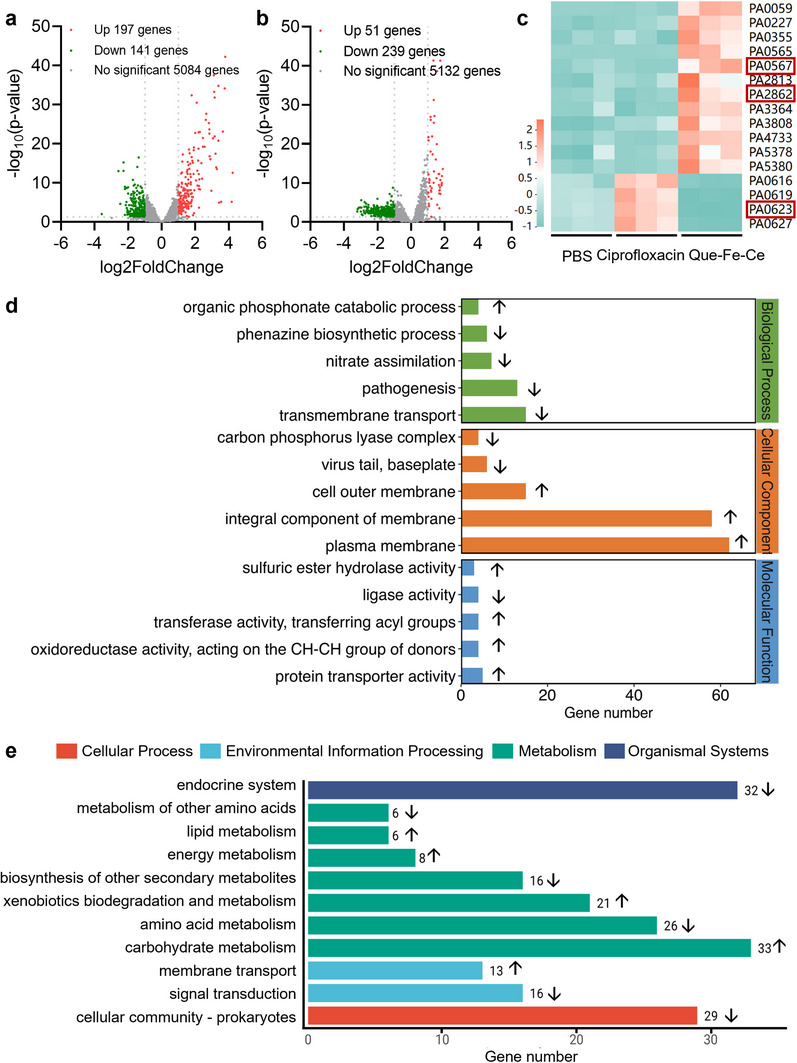
Transcriptomic analysis of *P. aeruginosa* exposed to Que‐Fe‐CeMPF or ciprofloxacin. a) Volcano plot of differentially expressed genes in *P. aeruginosa* exposed to Que‐Fe‐CeMPF compared to PBS. b) Same as a, but for *P. aeruginosa* exposed to ciprofloxacin compared to PBS. c) Heat map of mainly differential genes in *P. aeruginosa* exposed to Que‐Fe‐CeMOF or ciprofloxacin compared to PBS. d) GO pathway analysis and e) KEGG pathway analysis of differentially expressed genes of *P. aeruginosa* exposed to Que‐Fe‐CeMPF, compared to PBS. ↑ indicates upregulation; ↓ indicates downregulation.

The effect of Que‐Fe‐CeMPF and ciprofloxacin on gene expression of *P. aeruginosa* was explored by RNA‐Sequencing. Compared to the PBS group, 6.2% (338 genes) and 5.4% (290 genes) of the total 5422 genes were differentially regulated upon exposure to Que‐Fe‐CeMPF and ciprofloxacin, respectively (**Figure** **4**a,b). When exposed to ciprofloxacin, only 51 genes were upregulated, whereas exposure to Que‐Fe‐CeMPF led to 197 upregulated genes, which were mainly involved in integral components of membrane genes, as, e.g. *PA0567* and *PA2862*.^[^ ^29^, ^30^ ^]^ The downregulated genes were mainly involved in viral genome processes, particularly in relation to viral genome ejection through the host cell envelope, as, e.g., the *PA0623* gene.^[^ ^31^ ^]^ However, the gene of *PA0623* is upregulated in *P. aeruginosa* when exposed to ciprofloxacin (Figure 4c). The results of GO enrichment analysis indicated that differentially expressed genes in the Que‐Fe‐CeMPF group were primarily associated with cellular components, such as the plasma membrane, integral component of the membrane, and the outer cell membrane (Figure 4d). In contrast, differential gene expression in *P. aeruginosa* exposed to ciprofloxacin was enriched in both cellular components and biological processes (Figure S6a, Supporting Information). Additionally, KEGG pathway analysis indicated that Que‐Fe‐CeMPF‐exposure resulted in alterations in metabolic pathways in *P. aeruginosa* compared to the PBS control (Figure 4e). Notably, there were no changes in drug‐resistance‐related pathways in the Que‐Fe‐CeMPF group, while ciprofloxacin exposure resulted in differential gene expression involved in drug‐resistance pathways (Figure S6b, Supporting Information).

### 2.5 Biofilm Eradication by Que‐Fe‐CeMPF In Vitro

**Figure 5:**
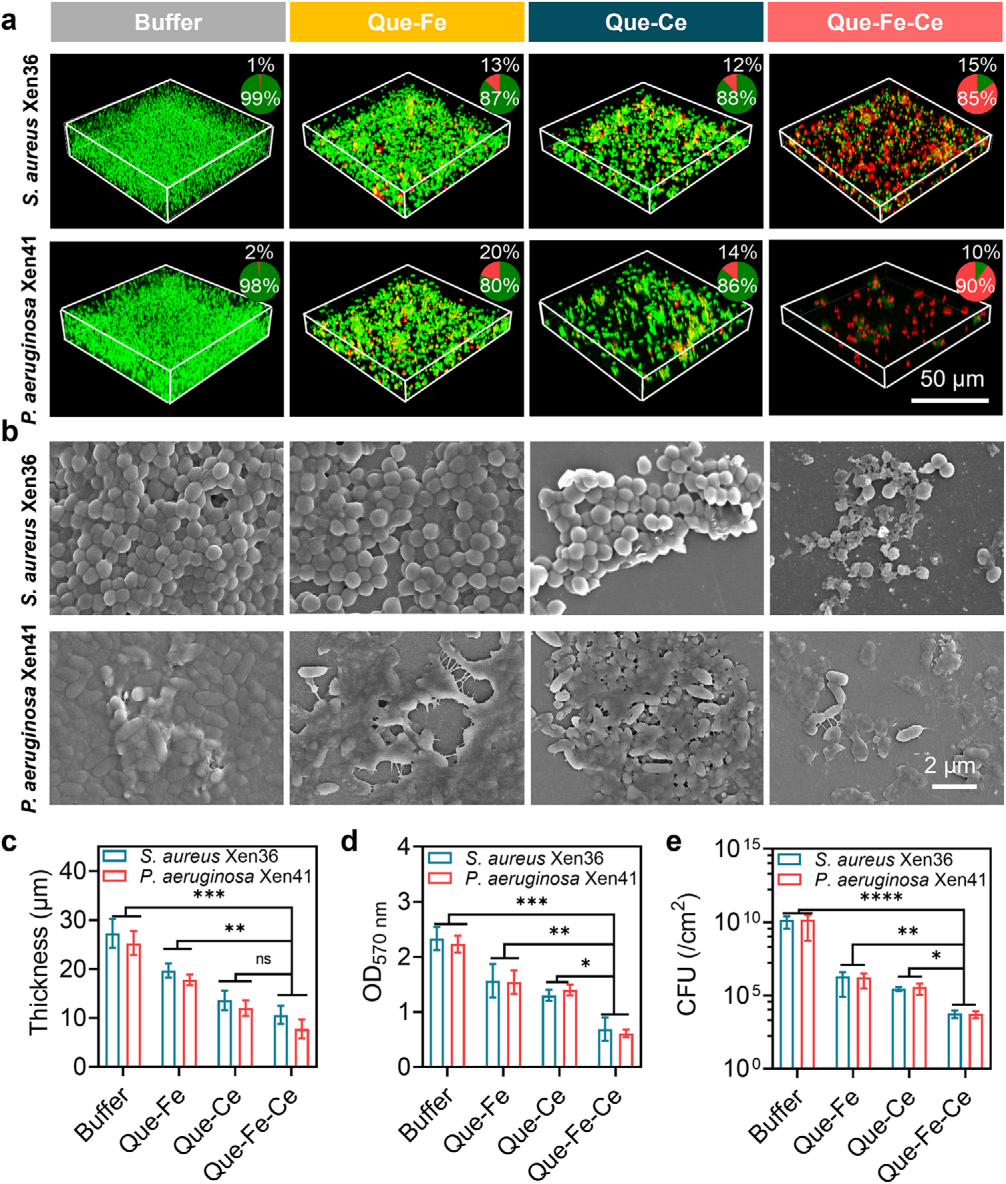
Antibiofilm activity of Que‐FeMPF, Que‐CeMPF, and Que‐Fe‐CeMPF in sodium acetate buffer at pH 4.5 in vitro. a) Representative 3D CLSM images of live/dead stained *S. aureus* and *P. aeruginosa* biofilms exposed to 50 µm MPFs for 3 h in the presence of H_2_O_2_ (100 µm), green fluorescent are live bacteria, and red fluorescent are cell wall‐damaged. The inset pie charts show the corresponding percentage of green/red fluorescence intensity in the biofilms. b) Same as panel a, now representative SEM images of *S. aureus* and *P. aeruginosa* biofilms. c) Biofilms thickness, derived from 3D CLSM images as presented in panel a. d) Biomass measured by the OD_570nm_ values of crystal violet stained biofilms under the same conditions as panel a. e) The CFUs of *S. aureus* and *P. aeruginosa* per cm^2^ in biofilms determined by plate counting under the same conditions as panel a. *p < 0.05 and **p < 0.01, ***p < 0.001, and ****p < 0.0001 indicates statistical significance (one‐way ANOVA) over the differences indicated by the spanning bars.

Exposure of biofilms to Que‐Fe‐CeMPF in the presence of H_2_O_2_ yielded higher killing efficiency for *S. aureus* and *P. aeruginosa*, compared to Que‐Fe and Que‐Ce MPFs (**Figure** **5**a). SEM analysis revealed complete destruction of the biofilms when exposed to Que‐Fe‐CeMPF in the presence of H_2_O_2_, while a robust and dense biofilm remained intact and fully covered the glass surface when exposed to buffer (Figure 5b). The biofilm structure became more open after exposure to Que‐Fe‐CeMPF compared to either Que‐FeMPF or Que‐CeMPF, which is due to the degradation of the biofilm matrix (Figure S7, Supporting Information). Reductions in biofilm thickness for *S. aureus* and *P. aeruginosa* (Figure 5c), along with decreases in biomass (Figure 5d), and CFU counts (Figure 5e), were significantly more pronounced when biofilms were exposed to Que‐Fe‐CeMPF than to Que‐FeMPF and Que‐CeMPF. These results demonstrate the catalytic role of the Ce^4+^ and Fe^3+^ ions immobilized in Que‐Fe‐CeMPF, which accelerates biofilm dispersal.

### 2.6 Biosafety of Que‐Fe‐CeMPF

The biosafety of Que‐Fe‐CeMPF was evaluated by measuring cell viability of fibroblasts, endothelial cells (HUVEC), and hemolysis of red blood cells exposed to Que‐Fe‐CeMPF in vitro. The metabolic activity expressed as cell viability of the fibroblasts and endothelial cells was found to be concentration dependent, with a viability > 80% when the Que‐Fe‐CeMPF was < 1.6 mm (Figure S8a,c, Supporting Information). Both fibroblasts and endothelial cells showed 100% viability when exposed to 50 µm of Que or the MPFs (Figure S8b,d, Supporting Information). No hemolysis of red blood cells from mice was observed in the presence of different MPFs and Que (Figure S9a, Supporting Information), even when Que‐Fe‐CeMPF was used at concentrations as high as 3.2 mm (Figure S9b, Supporting Information).

### 2.7 Inflammation Modulation of Que‐Fe‐CeMPF In Vitro

**Figure 6:**
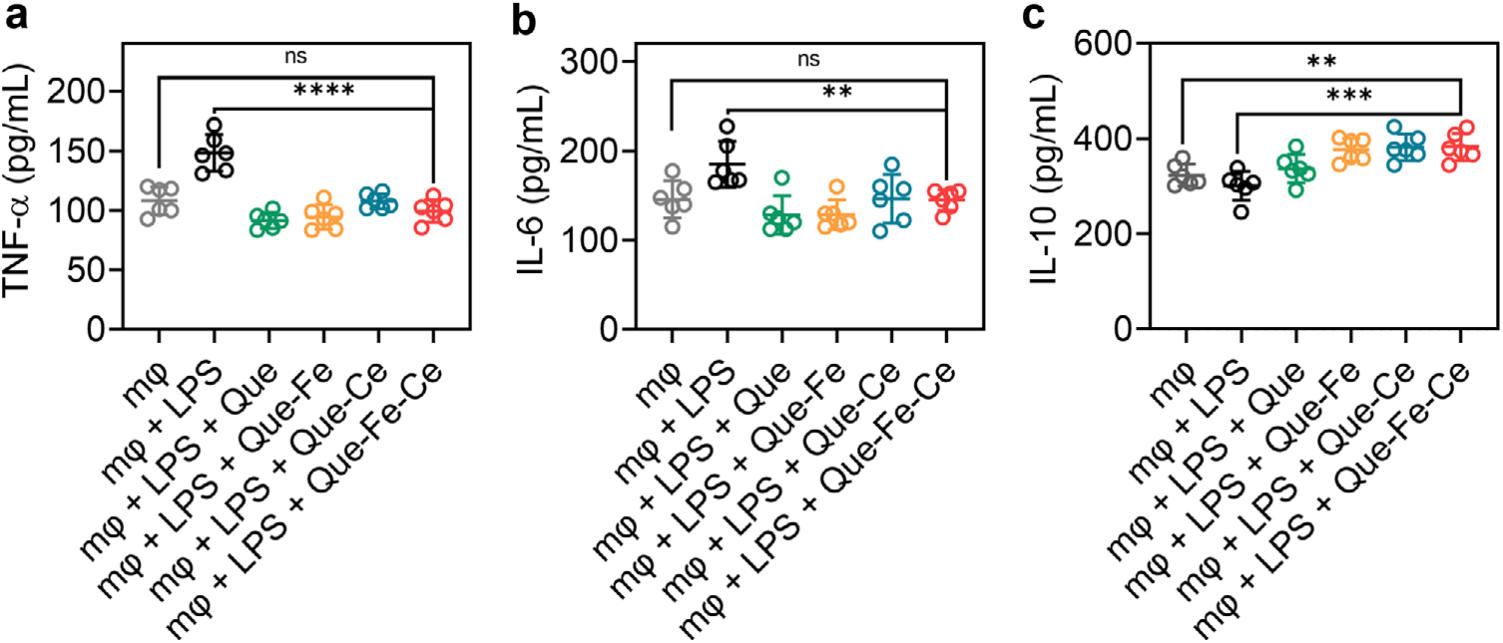
Cytokine expression by macrophages (mφ) after different treatments. a) TNF‐α expression measured using an enzyme‐linked immunosorbent assay. Nonactivated macrophages were used as the negative control, and LPS‐activated macrophages as the positive control. b) Same as panel a, now for IL‐6 secretion, and c) same as panel a, now for IL‐10 secretion. The concentrations of TNF‐α, IL‐6, and IL‐10 were determined using their standard curves (Figure S10, Supporting Information). **p < 0.01, ***p < 0.001, and ****p < 0.0001 indicate statistical significance (one‐way ANOVA) over the differences indicated by the spanning bars.

The effect of Que‐Fe‐CeMPF on inflammatory cytokine production by macrophages was investigated by measuring the secretion of pro‐inflammatory cytokines, such as tumor necrosis factor‐α (TNF‐α) and interleukin‐6 (IL‐6), as well as the anti‐inflammatory cytokine interleukin‐10 (IL‐10). TNF‐α and IL‐6 are markers of polarization toward the M1 phenotype, while IL‐10 secretion is a characteristic of the M2 phenotype. Exposure of macrophages to LPS resulted in macrophage activation and an increase in TNF‐α, IL‐6, and a decrease in IL‐10. Exposure of activated macrophages to Que or MPFs in the presence of H_2_O_2_ led to a decrease in TNF‐α and IL‐6 to levels similar to those produced by nonactivated macrophages (**Figure** **6**a,b). Moreover, Que‐Fe‐CeMPF resulted in a significant increase in IL‐10 compared to nonactivated macrophages, indicating a shift toward the M2 phenotype (Figure 6c).

### 2.8 Treatment of *P. Aeruginosa* Infected Lung Tissue Using Que‐Fe‐CeMPF in a Pneumonia Mouse Model

The conclusions regarding the biosafety, antibacterial activity, and anti‐inflammatory effects of Que‐Fe‐CeMPF were based on in vitro experiments, showing that Que‐Fe‐CeMPF exhibited high antibacterial efficacy, reduced inflammatory factors, and had good biosafety at a concentration of 50 µM. For in vivo evaluation of antibacterial activity and reduction of the inflammation of Que‐Fe‐CeMPF, a murine pneumonia model was employed.

**Figure 7:**
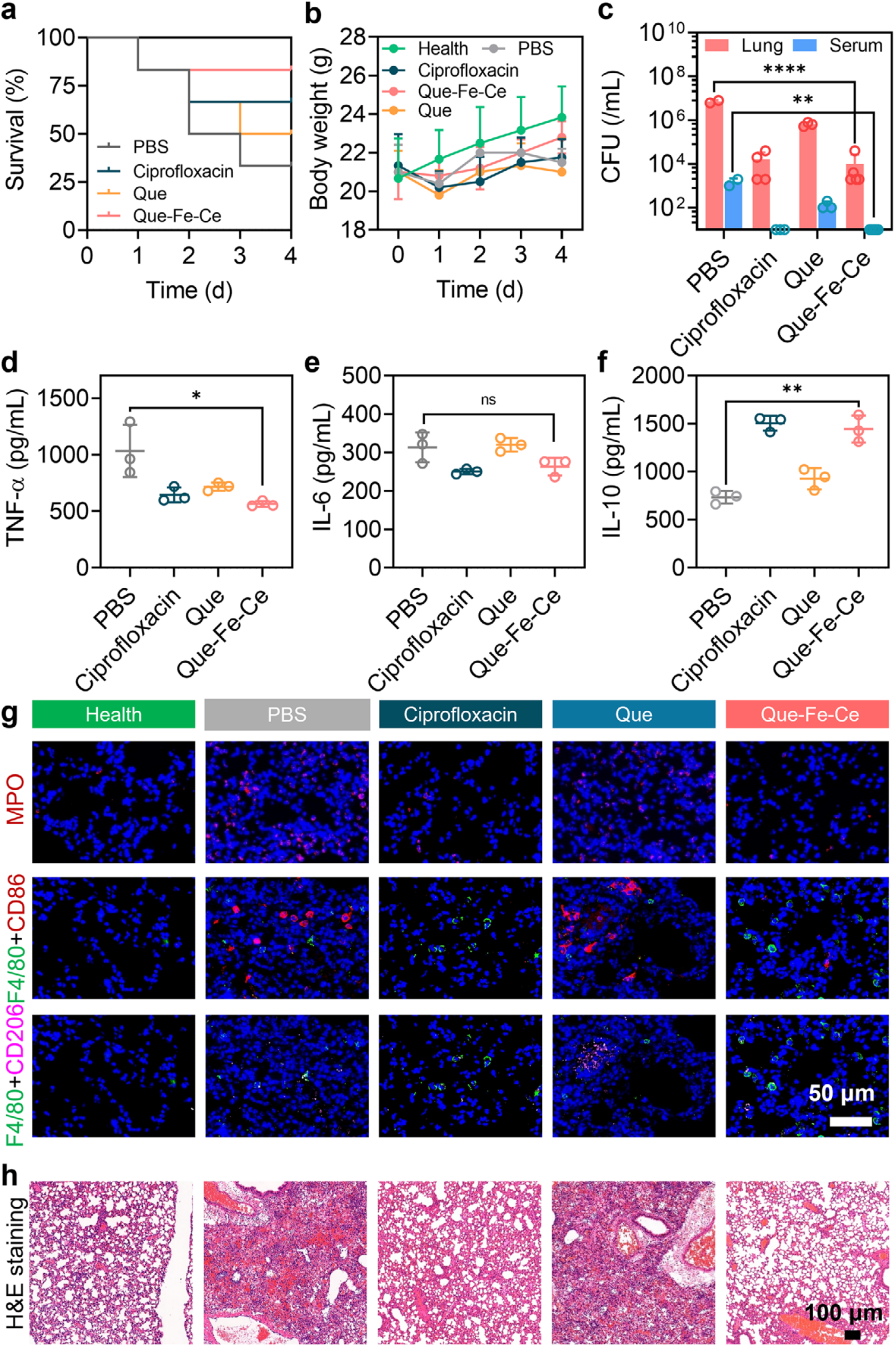
Therapeutic efficacy of Que‐Fe‐CeMPF in murine *P. aeruginosa* pneumonia model. a) Percent survival of the *P. aeruginosa‐*infected mice as a function of time after different treatments (*n* = 6). b) The body weight of the *P. aeruginosa‐*infected mice as a function of time after different treatments. c) CFUs of *P. aeruginosa* from lungs and serum of mice after different treatments. d) Secretion of TNF‐α (pro‐inflammatory cytokine), e) IL‐6 (pro‐inflammatory cytokine), and f) IL‐10 (anti‐inflammatory cytokine) in the lung at post‐treatment. g) Immunofluorescent images of lung tissue. Nuclei were stained with DAPI (blue) in all samples. The top row shows staining for myeloperoxidase (MPO, purple), a marker for neutrophils. Macrophages were labeled with F4/80 (green). To differentiate between macrophages, CD86 (red) was used as a marker for M1 macrophages and CD206 (pink) as a marker for M2 macrophages. Note: CD86 and CD206 staining were performed on the same tissue sample, but the corresponding images are shown separately due to the difficulty in distinguishing red and pink signals in a single image. h) H&E staining images of lung tissue at post‐treatment. *p < 0.05 and **p < 0.01 indicate statistical significance (one‐way ANOVA) over the differences indicated by the spanning bars.

On day four, after three treatments, the survival rates of the mice were as follows: 83.3% in the Que‐Fe‐CeMPF treated pneumonia group, 66.7% in the ciprofloxacin group (positive control), 50% in the Que group, and 33.3% in the PBS group (negative control) (**Figure** **7**a). All infected mice had a 10–15% reduction in body weight compared to that of the healthy mice (Figure 7b). After sacrificing the mice, the number of *P. aeruginosa* CFUs in the lung tissue of the ciprofloxacin and Que‐Fe‐CeMPF‐treated mice was similar and showed a decrease of 3 log units compared to the PBS group (Figure 7c). No bacteria were detected in the blood. The CFU count in blood for the PBS and Que‐treated mice indicated a potential for sepsis (Figure 7c). For cytokine levels, ciprofloxacin, Que, and Que‐Fe‐CeMPF treatments all showed comparable reductions in TNF‐α compared to the PBS‐treated mice, with no significant differences for the IL‐6 (Figure 7d,e). However, ciprofloxacin and Que‐Fe‐CeMPF induced a twofold increase in IL‐10 compared to PBS and Que (Figure 7f).

In addition to the cytokine determination, immunofluorescence staining was used to study macrophage polarization in the lungs (Figure 7g). Mice treated with ciprofloxacin and Que‐Fe‐CeMPF exhibited lower lung myeloperoxidase (MPO) levels, which is a measure of inflammation, than the PBS and Que groups. Immunofluorescence images of lung tissue showed stronger red fluorescence (CD86, expressed on M1 phenotype macrophage) in the PBS and Que‐treated mice, indicating macrophage activation and a shift toward the pro‐inflammatory M1 phenotype. In contrast, the Que‐Fe‐CeMPF and ciprofloxacin‐treated mice showed stronger pink fluorescence (CD206, indicating M2 phenotype macrophage), suggesting a shift toward the anti‐inflammatory M2 phenotype in vivo. Hematoxylin‐eosin (H&E) staining of the lungs showed numerous intact and hollow alveoli in the Que‐Fe‐CeMPF‐treated mice, similar to healthy mice. In contrast, the ciprofloxacin‐treated mice had worse lung conditions compared to healthy mice; fusion and incomplete inflation of alveoli were observed in the PBS and Que groups (Figure 7h). No significant changes were observed in other major organs (heart, liver, spleen, and kidney) of infected mice across all groups (Figure S11, Supporting Information). Blood biochemistry showed no difference between the treatment groups and healthy mice (Figure S12, Supporting Information). However, the number of white blood cells (WBC) and relevant hemoglobin parameters (MCH, MCV, and MCHC) were higher in the PBS group compared to healthy mice and other treatment groups, likely due to the bacterial infection.

## 3 Discussion

In this study, a novel strategy was proposed to combat drug‐resistant bacterial infections, focusing on biofilm dispersal, bacterial killing, and modulation of the host immune response at the infection site. This multimodal approach was realized through the development of the bimetallic‐phenolic framework, Que‐Fe‐CeMPF. Que‐Fe‐CeMPF exhibits enhanced antibacterial activity compared to the single‐metal phenolic frameworks (Que‐FeMPF and Que‐CeMPF), particularly under acidic conditions (Figure 3; Figure S5, Supporting Information). Moreover, the incorporation of the second metal ion does not affect the zeta potential or morphology of Que‐Fe‐CeMPF (Figure S1, Supporting Information). Que‐Fe‐CeMPF is able to hydrolyze the biofilm matrix by its nuclease‐mimetic activity, facilitating biofilm dispersion. The degradation of the biofilm allowed Que‐Fe‐CeMPF to penetrate more deeply into the biofilm, thereby increasing its bacterial killing efficiency. Additionally, Que‐Fe‐CeMPF accelerates Fenton reactions, enhancing ·OH generation in acidic environments. These radicals damage bacterial cell walls, leading to the loss of intracellular protein and ultimately, bacterial cell death.

Que‐Fe‐CeMPF is targeting bacteria through the phenolic hydroxyl groups, in an acidic environment, which can form hydrogen bonds with polysaccharides, membrane proteins, and peptidoglycan from the bacterial cell wall (Figure 3b). This interaction shortens the distance between the bacteria and Que‐Fe‐CeMPF, thereby improving the efficiency of ·OH delivery to the bacterial cells. The effectiveness of ·OH in killing bacteria is closely related to its short life‐time (3–5 µs), making it essential that the distance between the ·OH source and the bacteria is minimized.^[^ ^32^ ^]^ The short lifetime of ·OH, along with its generation in a low pH environment, ensures biosafety to healthy tissues at normal pH. Another important advantage of Que‐Fe‐CeMPF is its low potential to induce drug‐resistance in contrast to ciprofloxacin (see Figure 4) and other conventional antibiotics, which target specific bacterial components and are therefore more susceptible to resistance development.^[^ ^33^ ^]^ This resistance‐avoidance is attributed to the multi‐target mechanism of Que‐Fe‐CeMPF, which makes it more difficult for bacteria to develop resistance.

The emergence of metal nanomaterials holds great promise as potential agents in the fight against drug‐resistant bacterial infections. Oxidative stress induced by reactive oxygen species (ROS) is a key mechanism supporting the antibacterial activity of metal nanomaterials.^[^ ^34^ ^]^ However, this mechanism also carries the risk of non‐specific cytotoxicity, potentially harming healthy tissue cells. Therefore, achieving precise control over ROS generation at infection sites is essential to maximize pathogen‐selective oxidative damage while minimizing collateral injury to healthy tissues. Infected sites are typically acidic and rich in hydrogen peroxide (H_2_O_2_) due to the host immune response. Neutrophils and macrophages generate ROS, including H_2_O_2_, during the oxidative burst to kill pathogens, while increased metabolic activity, hypoxia, and acidic byproducts from both immune and bacterial cells contribute to local acidification. By leveraging the pH difference between infected and healthy tissues, it becomes possible to selectively control ROS production within the acidic microenvironment of infected tissues.^[^ ^35^ ^]^ Fe^2^⁺ exhibits high catalytic activity specifically under acidic conditions, where it efficiently catalyzes the conversion of H_2_O_2_ into ·OH via Fenton reaction, outperforming other metal ions like Cu^2^⁺ or Mn^2^⁺.^[^ ^36^ ^]^ However, naturally low H_2_O_2_ levels (≈100 µm) limit ·OH production, while externally administration of H_2_O_2_ carries the risk of damaging healthy tissues. Therefore, enhancing the low‐concentration H_2_O_2_ to ·OH is critical for optimizing the balance between antibacterial efficacy and biological safety.

Unlike some previous studies, where the conversion of H_2_O_2_ to ·OH is activated through light stimulation,^[^ ^37^ ^]^ the Que‐Fe‐CeMPF system exhibits intrinsic catalytic activity under acidic conditions, without requiring light. Light‐dependent strategies are often limited by shallow tissue penetration and spatially restricted irradiation. Here, Que accelerates Fe^3^⁺→Fe^2^⁺ generation, thereby sustaining catalytic chain reactions (Figure 2a). The MPF's nanonetworks further promote H_2_O_2_ adsorption and its subsequent conversion into ·OH via a large surface area. Additionally, catalytic activity significantly decreases when the pH increases from 4.5 to 7.4, enabling selective ·OH generation in the acidic microenvironment of infection sites, while minimizing cytotoxicity in healthy tissue (Figure 2c). This pH‐dependent catalytic specificity is crucial for achieving both therapeutic efficacy and biological safety in ROS‐based antibacterial strategies.

Biofilms pose a significant barrier to effective antibacterial treatment by impeding drug penetration and reducing therapeutic efficacy. eDNA, a vital structural component of the EPS in biofilms, plays a critical role in maintaining the stability and integrity of the biofilm matrix, making it a compelling and strategic target for biofilm dispersal.^[^ ^38^ ^]^ However, the phosphodiester bonds within eDNA exhibit substantial resistance to hydrolysis under neutral pH conditions,^[^ ^39^ ^]^ presenting a major challenge for biofilm dispersal. Although DNase, a natural enzyme capable of DNA hydrolysis activity, can effectively degrade eDNA, its enzymatic activity is limited in the acidic microenvironment of biofilms.^[^ ^40^ ^]^ In recent years, nanozymes have emerged as promising alternatives, with nuclease‐mimetic capabilities enabled by acidic Lewis metal ions such as Ce^4+^, Zn^2+^, and Mg^2+^.^[^ ^41^ ^]^ Among these, Ce complexes exhibit superior catalytic activity, surpassing that of traditional Zn and Mg‐based molecular mimetics.^[^ ^42^ ^]^ Unlike core‐shell bimetallic nanomaterials, where the function of the core metal is delayed until the shell degrades, the Fe^3+^ and Ce^4+^ ions in Que‐Fe‐CeMPF operate synergistically while maintaining their individual catalytic roles. The synergistic interaction enhances the catalytic efficiency of Que‐Fe‐CeMPF for biofilm matrix hydrolysis, making it a potent and attractive agent for biofilm dispersal.

Immune regulation is crucial for tissue repair at infection sites once the infection is controlled by the immune system or exogenous drugs. M2 macrophages promote angiogenesis and vascular maturation to accelerate tissue repair after pathogen clearance.^[^ ^43^ ^]^ However, after bacterial infection, the transition from pro‐inflammatory M1 phenotype to anti‐inflammatory M2 phenotype is often delayed.^[^ ^44^ ^]^ In this study, Que‐Fe‐CeMPF effectively stimulated macrophage polarization toward the “repairing” M2 phenotype by upregulating the anti‐inflammatory cytokine IL‐10 and downregulating pro‐inflammatory cytokines TNF‐α and IL‐6 (Figure 7). Macrophage polarization from M1 to M2 has also been achieved using copper‐based metal‐organic frameworks embedded with Ag to accelerate wound healing.^[^ ^45^ ^]^ Similarly, NO production through a near‐infrared‐triggered NO release system has been applied to accelerate healing in MRSA‐infected diabetic wounds.^[^ ^46^ ^]^ However, such NIR‐dependent approaches are constrained by limited tissue penetration and the need for external irradiation, which reduces their clinical applicability. In contrast, Que‐Fe‐CeMPF enables immune modulation without the need for light activation, offering a more practical therapeutic option. While other approaches have focused on stimulating M1 polarization to combat bacterial infections,^[^ ^47^, ^48^ ^]^ clinical priorities often shift toward tissue regeneration once the bacterial burden is controlled. Effective infection management should therefore involve not only the eradication of pathogens but also the resolution of inflammation and stimulation of tissue repair. Que‐Fe‐CeMPF uniquely addresses these multifaceted therapeutic needs by combining potent antibacterial activity with the ability to modulate immune responses and promote healing, making it a promising agent for treating complex, drug‐resistant bacterial infections. The survival rate of mice with pneumonia infection did not reach 100% survival under the treatment conditions used. To improve the survival rate, all bacteria in the biofilm at the infection site need to be killed, which probably requires an extended treatment period.

## 4 Conclusion

A bimetal‐phenolic framework with multimodal activity for bacterial killing, biofilm degradation under acidic conditions and inflammation modulation was successfully designed. Que‐Fe‐CeMPF catalyzes the generation of ·OH from H_2_O_2_, which disrupts the bacterial cell wall, leading to intracellular protein leakage and bacterial death. Moreover, Que‐Fe‐CeMPF modulates the immune response by regulating cytokine levels, inducing macrophages to adapt a more M2‐like phenotype, enhancing immune regulation, and promoting tissue repair at the site of bacterial infection. In vivo treatment of *P. aeruginosa* induced pneumonia in a mouse model demonstrated faster bacterial killing and lung tissue recovery with Que‐Fe‐CeMPF compared to free Que and the antibiotic ciprofloxacin. Additionally, unlike ciprofloxacin, which directly induces drug‐resistance, Que‐Fe‐CeMPF did not affect drug‐resistance pathways, making it less likely to promote the development of drug‐resistance in bacteria. Therewith, this work demonstrates that the multimodal activity of the Que‐Fe‐CeMPF effectively controls drug‐resistant bacterial infections and accelerates healing in vivo.

## 5 Experimental Section

### Chemicals and Materials

Quercetin, ferric chloride hexahydrate (FeCl_3_·6H_2_O), ammonium cerium nitrate (Ce(NH_4_)_2_(NO_3_)_6_), and ethylene diamine tetraacetic acid (EDTA) were purchased from Macklin (Shanghai, China). H_2_O_2_ (30%), agarose, dimethyl sulfoxide (DMSO), silicon dioxide (SiO_2_), and LPS (Lipopolysaccharides from *Escherichia coli* O111:B4, S1732) were obtained from Sigma‐Aldrich (Shanghai, China). 3,3′,5,5′‐Tetramethylbenzidine (TMB), tetracycline, kanamycin, crystal violet, and NH_2_C(CH_2_OH)_3_ (Tris base) were purchased from Aladdin (Shanghai, China). 2′,7′‐dichlorofluorescein diacetate (DCFH‐DA), 4% paraformaldehyde (tissue fixative), streptomycin, and the bicinchoninic acid (BCA) test kit were obtained from Solarbio (Beijing, China). LIVE/DEAD bacterial staining kit (SYTO 9, propidium iodide), TNF‐α, IL‐6, and IL‐10 ELISA kits were provided by Invitrogen (Shanghai, China). Cell counting kit‐8 (CCK‐8) was provided by Biosharp (Beijing, China). Phosphate‐buffered saline (PBS, 5 mm K_2_HPO_4_, 5 mm KH_2_PO_4_, 150 mm NaCl, pH 7.4), sodium carbonate buffer (0.8 mm Na_2_CO_3_, 142.8 mm NaHCO_3_, pH 8.5), and sodium acetate buffer (23 mm CH_3_COONa, 28 mm CH_3_COOH, pH 4.5) were provided by Coolaber (Beijing, China). Tryptic Soy broth (TSB) and Luria‐Bertani broth (LB) were obtained from Hangzhou Microbial Reagent Co., Ltd. (Hangzhou, China). RPMI‐1640 medium and DMEM‐HG were purchased from ThermoFisher Scientific, Inc. (Carlsbad, USA). Endothelial cell medium (ECM) and endothelial cell growth supplement (ECGS) were purchased from ScienCell (Carlsbad, USA). Fetal bovine serum (FBS) was purchased from Gibco (Shanghai, China). Penicillin was obtained from Genview (Beijing, China). RNAprep pure Cell/Bacteria Kit and TIANSeq rRNA Depletion Kit were provided by TIANGEN (Beijing, China). All chemicals were used without further purification.

### Preparation of Que‐Fe‐CeMPF

Que‐Fe‐CeMPF was synthesized through a direct assembly process of quercetin and metal ions (Fe and Ce ions).^[^ ^49^ ^]^ Briefly, quercetin (Que) in DMSO (100 µL, 60 mm) was dripped into sodium carbonate buffer (9.8 mL, pH 8.5) and stirred for 10 min at room temperature. Then Ce(NH_4_)_2_(NO_3_)_6_ in ultrapure water (100 µL, 10 mm) was added dropwise to the above reaction mixture and stirred for 30 min at room temperature. Next, FeCl_3_·6H_2_O in ultrapure water (100 µL, 10 mm) was added dropwise to the mixture and stirred for 12 h at room temperature. After this, the Que‐Fe‐CeMPF was dialyzed (molecular weight cutoff, 500 Da) against water for 48 h, with water refreshed twice during dialysis. After purification, the Que‐Fe‐CeMPF was freeze‐dried and stored at room temperature in a desiccator with SiO_2_. The syntheses of Que‐FeMPF and Que‐CeMPF were performed in a similar manner, but with only one metal ion (Fe^3+^ or Ce^4+^) added, respectively.

### Characterizations of Que‐Fe, Que‐Ce, and Que‐Fe‐Ce MPFs

Zeta potentials of 10 µm Que‐FeMPF, 10 µm Que‐CeMPF, and 10 µm Que‐Fe‐CeMPF in PBS (pH 7.4) were measured using the Malvern Zetasizer Nano ZS. The morphologies of the MPFs were characterized using field‐emission scanning electron microscopy (FESEM, SU8010, Hitachi, Japan). The MPFs suspended in ultrapure water were placed on conductive tape, dried at room temperature, and sputter‐coated with gold for SEM imaging. Transmission electron microscopy (TEM, FEI Talos 200s, Thermo Fisher Scientific, USA) was used to characterize the morphology of Que‐Fe‐CeMPF. High‐angle annular dark‐field (HAADF) and energy dispersive X‐ray spectroscopy mapping of TEM were used to determine the elemental distribution in Que‐Fe‐CeMPF. For TEM analysis, a suspension of 10 µm Que‐Fe‐CeMPF was dropped onto a carbon‐coated copper grid and dried slowly in vacuo at room temperature.

X‐ray photoelectron spectroscopy (XPS, Kratos Analytical Ltd., Axis Ultra DLD, Germany) was used to measure the elemental surface composition of Que‐Fe‐CeMPF. The MPF was pressed into a tablet and then taped to the sample holder. X‐rays were produced using an Al‐coated anode with a spot size of 400 µm. Wide scans were taken over the binding energy range of 0–1200 eV with a pass energy of 150 eV. High resolution narrow scans were taken with a pass energy of 50 eV for Fe 2p (707–733 eV) and Ce 3d (875–925 eV). Fourier transform infrared spectroscopy (FT‐IR, Bruker, Tensor II, Germany) was used to determine the functional groups of Que‐Fe‐CeMPF and Que powder. The MPF was mixed with KBr (1:100 weight ratio) and pressed into a tablet. A blank KBr tablet was used as the background. The FT‐IR spectra were recorded over the wavenumber range of 4000–400 cm^−1^ with a resolution of 0.4 cm^−1^, and 16 scans were taken and averaged.

### Catalytic Activity of Que‐Fe, Que‐Ce, and Que‐Fe‐Ce MPFs

The catalytic activity of the synthesized MPFs was determined using 3,3′,5,5′‐tetra‐methylbenzidine (TMB).^[^ ^50^ ^]^ Briefly, 30 µL of TMB in DMSO (20 mm) was mixed with 2.64 mL of sodium acetate buffer (pH 4.5), 30 µL of H_2_O_2_ in ultra‐pure water (10 mm, final concentration 100 µm), and 300 µL Que‐Fe, Que‐Ce, or Que‐Fe‐Ce MPFs (500 µm, final concentration 50 µm) in ultra‐pure water. The mixture was incubated in the dark at 37 °C for 10 min. Then 200 µL of the reaction mixture was transferred to a clean 96‐well plate, and the absorbance was measured using a UV‐2600 spectrophotometer (Shimadzu, Japan) at 37 °C. The absorbance peak at 652 nm was used to quantify the catalytic activity of the synthesized MPFs. The catalytic activity of metal ions (Fe^3+^, Ce^4+^, Fe^3+^ + Ce^4+^), Que, and Que directly mixed with metal ions was also determined using the same procedure.

The catalytic kinetics of Que‐Fe‐CeMPF were also measured with different concentrations of H_2_O_2_ (0, 0.5, 1, 1.5 and 2 mm) to determine the apparent kinetic parameters. These parameters were calculated using the Michaelis‐Menten equation:

(1) $$V=V_{\max}\;\times \;\left[S\right]/\left(K_{m}+\left[S\right]\right)$$

where *V* is the reaction velocity, *V_max_* is the maximal reaction velocity, [S] is the concentration of H_2_O_2_, and *K_m_* is the Michaelis constant.

The reaction rate *V* was calculated using the equation:

(2) V=Δc/Δt

According to the Beer–Lambert law:

(3) Abs=ε·c·l

where Abs is the UV–vis absorbance of the oxidized TMB at 652 nm, *l* = 1 cm was the optical path length, and *ε* = 39000 M^−1^⋅cm^−1^ was the molar extinction coefficient, and c could be calculated.

Finally, to study the stability of the catalytic activity of Que‐Fe‐CeMPF, its catalytic performance was evaluated under varying conditions: at different temperatures (25, 37, 40, 50, 60, and 70 °C), in buffers with different pH levels (sodium acetate at pH 3.5, pH 4.5, and pH 5.5; phosphate buffered saline at pH 7.4; and sodium carbonate at pH 8.5) and after different storage durations in a desiccator with SiO_2_ at room temperature (1, 3, 5, 7, and 21 day).

### Bacterial Growth Conditions and Harvesting

*S. aureus* Xen36 and *P. aeruginosa* Xen41 (PerkinElmer Inc., Waltham, MA, USA) were cultured in 10 mL tryptone soya broth (TSB) supplemented with 100 µg mL^−1^ kanamycin or 10 mL Luria‐Bertani broth (LB) supplemented with 60 µg mL^−1^ tetracycline, respectively, at 37 °C for 24 h. Afterward, the preculture was transferred to 200 mL growth media containing either kanamycin or tetracycline and cultured for another 18 h. The bacterial cultures were harvested by centrifugation at 3000 g for 5 min, washed twice with PBS, and resuspended in PBS. The bacterial concentration was determined using a Bürker‐Türk counting chamber.

### Influence of Que‐Fe‐CeMPF with and Without H_2_O_2_ on Planktonic Bacteria

*S. aureus* Xen36 or *P. aeruginosa* Xen41 (2×10^7^ bacteria/mL, 100 µL) suspensions were added to a 96‐well plate, followed by an equal volume (100 µL) of Que‐Fe‐CeMPF at various concentrations (0, 25, 50, 100, and 200 µm) in sodium acetate buffer (pH 4.5), with or without H_2_O_2_ (200 µm). The final concentration of H_2_O_2_ is 100 µm. The plate was left for 3 h at 37 °C. After incubation, the bacterial solution was serially diluted in PBS, spread onto TSB agar plates for *S. aureus* and LB agar plates for *P. aeruginosa*, and incubated at 37 °C for 18 h. The CFUs were then counted. In a parallel experiment, the antibacterial activity of different MPFs suspensions (50 µm Que, Que‐Fe, Que‐Ce, or Que‐Fe‐Ce) was determined by CFU enumeration at pH 4.5 or 7.4 in the presence of hydrogen peroxide (100 µm). The procedure was the same as described for Que‐Fe‐CeMPF.

### Influence of MPFs on Bacterial Morphology by Scanning Electron Microscopy (SEM)

A silicon wafer (0.4 cm × 0.4 cm) was placed in a 96‐well plate. Then, 100 µL of *S. aureus* Xen36 or *P. aeruginosa* Xen41 (2 × 10^7^ bacteria/mL) suspended in sodium acetate buffer pH 4.5 was added, followed by 100 µL of MPFs (100 µm) suspended in sodium acetate buffer pH 4.5 with H_2_O_2_ (final concentration 100 µm). The plate was incubated at 37 °C for 3 h. Buffer alone was used as a control. After incubation, the silicon wafers were gently rinsed with sterile water to remove unattached bacteria, and the bacteria on the silicon wafers were then fixed with 4% paraformaldehyde for 15 min. After fixation, the bacteria were dehydrated in a series of ethanol solutions (30–100%) and dried in air. The attached bacteria on the silicon wafer surfaces were observed using a Field Emission Scanning Electron Microscope (SU8010, Hitachi, Japan) with an accelerating voltage of 5.0 kV.

### Killing Efficacy of the MPFs on Planktonic Bacteria

500 µL *S. aureus* Xen36 or *P. aeruginosa* Xen41 (2 × 10^7^ bacteria/mL) suspensions were exposed to 500 µL MPFs (100 µm) with H_2_O_2_ (200 µm) at pH 4.5 for 3 h in a confocal dish at 37 °C. The confocal dish was then gently rinsed with sterile water to remove unattached bacteria, and bacteria attached to the confocal dish were stained with LIVE/DEAD stain (1 µM SYTO9 and 1 µm propidium iodide) for 20 min in the dark. Green fluorescent stained bacteria indicate live cells, while red fluorescent bacteria indicate cell wall damage. After 20 min, the stain was removed, and the adhering bacteria were immersed in PBS for imaging using confocal laser scanning microscopy (CLSM, Nikon, A1, Japan). An argon ion laser at 488 nm was used to excite SYTO9 (green fluorescent), and a HeNe laser at 543 nm was used to excite propidium iodide (red fluorescent), collecting fluorescence at 500−535 nm (SYTO9) and 583−688 nm (propidium iodide).

### Bacterial Cell Wall Permeability

Suspensions of *S. aureus* Xen36 or *P. aeruginosa* Xen41 in PBS (2 mL, 2 × 10^7^ bacteria/mL) were mixed with 2 mL MPFs (100 µm) with H_2_O_2_ (200 µm) in a 15 mL sterile centrifuge tube. After 3 h incubation at 37 °C, these suspensions were centrifuged at 3000 *g* for 10 min, and the supernatants were collected for total protein determination using a bicinchoninic acid (BCA) test kit. The BCA assay is based on the reduction of Cu^2+^ to Cu^+^ by protein in an alkaline medium with colorimetric detection of the cuprous cation (Cu^+^) by bicinchoninic acid, turning from green to purple (λmax = 562 nm). Absorbance at 562 nm was measured after 30 min at room temperature.

### Intracellular ROS Generation

The generation of intracellular reactive oxygen species (ROS) by Que‐Fe‐CeMPF was determined using the fluorescent ROS probe 2′,7′‐dichlorofluorescein diacetate (DCFH‐DA), through oxidation from the non‐fluorescent DCFH to the highly fluorescent DCF. DCFH‐DA was dissolved in DMSO and then diluted in PBS. 5 mL of DCFH‐DA (20 µm) in PBS was mixed with 5 mL *S. aureus* Xen36 and *P. aeruginosa* Xen41 (2 × 10^7^ bacteria/mL) in PBS. After incubation for 30 min in the dark while shaking, the bacteria were centrifuged and washed twice with PBS to remove any uninternalized DCFH‐DA. The bacteria were then suspended and diluted in PBS to a concentration of 2 × 10^7^ bacteria/mL. An aliquot of 100 µL of the bacterial suspension was put in a 96‐well plate, and 100 µL of the synthesized MPFs (100 µm) with H_2_O_2_ (200 µm) at pH 4.5 were added to the bacterial suspension. After incubation at 37 °C for 3 h in the dark, the fluorescence intensity (*Ex* = 488 nm*, Em* = 525 nm) was recorded on a Varioskan LUX microplate reader (ThermoFisher, USA).

### Transcriptome Analysis in P. Aeruginosa after Exposure to Que‐Fe‐CeMPF or Ciprofloxacin

*P. aeruginosa* was isolated from LB agar to obtain a single bacterial colony. Then, the colony was cultured in 100 mL LB with 50 µm Que‐Fe‐CeMPF and H_2_O_2_ (100 µm) or 0.25 µg mL^−1^ ciprofloxacin (0.5 MIC) at 37 °C for 12 h. PBS was used as the control group. Three replicates were prepared for each group. The bacteria were harvested by centrifugation at 3000 g for 5 min, washed twice with PBS, and then frozen in liquid nitrogen and quickly ground in a mortar. Total RNA was extracted from each sample using the RNAprep pure Cell / Bacteria Kit. RNA purity and quantity were assessed using a NanoDrop 2000 spectrophotometer (Thermo Scientific, USA). RNA integrity was assessed using the Agilent 2100 Bioanalyzer (Agilent Technologies, Santa Clara, CA, USA). Samples with qualified purity, quantity, and integrity were used for subsequent library construction. To construct strand‐specific RNA‐seq libraries, ribosomal RNA was removed using the TIANSeq rRNA Depletion Kit. Then they were sequenced on the Illumina sequencing platform (HiSeqTM 2500). Transcriptome sequencing and data analysis were carried out by OE Biotech Co., Ltd. (Shanghai, China).

Gene expression levels were calculated by RSEM software, and differential expression analysis was performed using DESeq2 (v1.4.5) with a P value threshold of ≤ 0.05 and a fold change ≥2. Significant terms and pathways were corrected by Q value and fold change by Bonferroni. The enrichment analysis of differently expressed genes was carried out using Gene Ontology (GO) and the Kyoto Encyclopedia of Genes and Genomes (KEGG) pathway. GO analyses included biological processes, cellular components, and molecular functions. KEGG analyses covered gene categories related to cellular processes, environmental information processing, human diseases, metabolism, and organismal systems.

### Biofilm Eradication of Que‐Fe‐CeMPF In Vitro

Bacterial biofilms were grown in 96‐well plates by adding 100 µL *S. aureus* Xen36 or *P. aeruginosa* Xen41 in PBS (10^8^ bacteria/mL) in a 96‐well plate and incubated at 37 °C for 1 h to allow bacterial adhesion. After removing the bacterial suspension, the well was washed once with PBS (100 µL). Then 200 µL of the appropriate growth medium was added, and the bacteria were incubated at 37 °C for 24 h to form biofilms. The growth medium was removed, and the biofilms were washed once with PBS (100 µL). Subsequently, the biofilms were exposed to 200 µL of MPFs (50 µm) in sodium acetate buffer pH 4.5 with H_2_O_2_ (100 µm) for 3 h. After treatment, the MPFs suspension was removed, and the biofilms were washed with 200 µL PBS. The biofilms were suspended in 200 µL PBS, serially diluted, and spread on agar plates. After incubation at 37 °C for 18 h, CFUs were counted. All experiments were done in triplicate using independently grown biofilms.

In a separate experiment, biofilm biomass after exposure to different MPFs was determined using crystal violet staining. Biofilms, grown as described above, were gently rinsed with PBS and air‐dried for 20 min. Next, 200 µL of crystal violet solution (1.0% w/v) was added to the biofilms. After 20 min incubation, the biofilms were rinsed with PBS and air‐dried for 10 min. To dissolve the crystal violet from the biofilm, 200 µL of 90% ethanol was added. 200 µL of the resulting solution was transferred to a fresh 96‐well plate, and the absorbance at 570 nm was recorded using a microplate reader (100‐240 VAC, USA). The biomass was determined in triplicate.

For analysis of *S. aureus* and *P. aeruginosa* biofilms with CLSM, bacteria were grown in confocal dishes. Briefly, 1 mL *S. aureus* Xen36 in TSB or *P. aeruginosa* in LB (10^8^ bacteria/mL) was added to a confocal dish and kept for 1 h at 37 °C to allow the bacteria to settle and adhere to the bottom of the confocal dish. The bacterial suspension was then removed, and the well was washed once with 1 mL PBS. 1 mL fresh growth medium was added to each confocal dish, and bacteria were incubated at 37 °C for 24 h. After incubation, the growth medium was removed, and the biofilms were washed once with 1 mL PBS. Then, the biofilms were exposed to 1 mL MPFs (50 µm) with H_2_O_2_ (100 µm) in sodium acetate buffer (pH 4.5) for 3 h. Buffer alone was used as a control. After 3 h, the MPFs suspensions were removed and the wells were washed once with PBS. The biofilms were stained with LIVE/DEAD stain (1 µm SYTO9 and 1 µm propidium iodide) for 20 min in the dark. After staining, the solution was removed and the wells were washed once with PBS. Finally, the biofilms were immersed in PBS and imaged using CLSM.

The morphologies of *S. aureus* and *P. aeruginosa* biofilms were analyzed by SEM. A silicon wafer (0.4 cm × 0.4 cm) was placed at the bottom of one well of a 96‐well plate. Bacterial biofilms were grown on silicon wafers as described above, and the biofilms were exposed to 200 µL of MPFs (50 µm) with H_2_O_2_ (100 µm) for 3 h. After treatment, the silicon wafers with biofilms were gently rinsed with sterile water to remove unattached bacteria, and the biofilms were fixed with 4% paraformaldehyde for 15 min. After fixation, the samples were dehydrated in a series of ethanol solutions (30–100%) and air‐dried. The silicon wafers were sputter‐coated with gold and observed using a Field Emission Scanning Electron Microscope (SU8010, Hitachi, Japan) at an accelerating voltage of 5.0 kV.

### Cleavage of the Biofilm Matrix

24 h‐old *S. aureus* Xen36 or *P. aeruginosa* Xen41 biofilms grown in a 24‐well plate were treated with 500 µL MPFs (50 µm) with or without H_2_O_2_ (100 µm) for 3 h in sodium acetate buffer pH 4.5. Subsequently, those biofilms were rinsed with PBS and dispersed in 1 mL PBS (pH 7.4). The biofilm suspensions were centrifuged at 3000 g for 15 min at 4 °C to collect the supernatants. A 1% agarose gel was prepared using 0.5 g agarose dissolved in 50 mL 1× TAE buffer (containing 0.74 g Tris base, 1.14 mL acetic acid, and 4.84 g EDTA per liter). The cleaved biofilm matrix products (10 µL) in the collected supernatants were determined using agarose gel (1%) electrophoresis and visualized using Gel Red staining. The resulting gel images were analyzed using a Gel Imager (Tanon3500BR, Shanghai, China).

### Biosafety of Que‐Fe‐CeMPF In Vitro

Mouse fibroblasts (L929) and human umbilical vein endothelial cells (HUVEC) were cultured in RPMI‐1640 medium supplemented with 10% fetal bovine serum, 1% penicillin/streptomycin, or in ECM supplemented with 5% fetal bovine serum, 1% ECGS, 1% penicillin/streptomycin (complete medium). Cell cultures were incubated at 37 °C with 5% CO_2_ and grown for 24 h. Afterward, cells were detached from the culture flask by trypsinization, collected by centrifugation at 1000 g for 5 min, and resuspended in fresh culture medium. Cell concentration was determined using a Bürker‐Türk counting chamber.

Cells were seeded in 96‐well plates (10^4^ cells/well) in complete medium and incubated for 24 h at 37 °C. Afterward, the growth medium was replaced with fresh complete medium containing various concentrations of Que‐Fe‐CeMPF (0.05 mM to 1.6 mm) and 50 µm of Que, Que‐FeMPF (at an equivalent Que concentration), or Que‐CeMPF (at an equivalent Que concentration). After incubation for another 24 h, cell metabolic activity was determined using the CCK‐8 assay. Briefly, the growth medium was removed, and 200 µL of fresh complete medium and 20 µL of the tetrazolium solution from the CCK‐8 kit were added to each well. Plates were incubated at 37 °C for 2 h. The tetrazolium salt was reduced by cellular dehydrogenases to form an orange formazan dye. Then, 200 µL of the solution was transferred to a fresh 96‐well plate, and the absorbance was recorded at 450 nm using a microplate reader. The experiment was performed in triplicate. Complete medium without cells served as the negative control (OD_medium_), and the cells exposed to complete medium as the positive control (OD_medium with cells_). The optical density of each experimental group was compared with the negative control. The relative cell viability (%) was calculated according to the following equation:

(4) $$\begin{array}{ccc} \mathrm{Cell}\;\mathrm{viability}\left(\%\right) & = & \left(OD_{\mathrm{MPF}}-OD_{\mathrm{medium}}\right)/\left(OD_{\mathrm{medium}\mathrm{with}\;\mathrm{cells}}\;-OD_{\mathrm{medium}}\right)\\ & & \times \;100\% \end{array}$$

Hemolysis of Que‐Fe‐CeMPF was evaluated using red blood cells (RBCs) obtained from healthy female ICR mice. Red blood cells (RBCs, 1 mL) were collected via cardiac puncture, harvested by centrifugation at 1000 g for 5 min at 4 °C, washed three times with PBS, and resuspended in 10 mL PBS. Then, 500 µL of RBC suspension was mixed with 500 µL of Que‐Fe‐CeMPF (at concentrations ranging from 0.1 to 12.8 mm), or 500 µL of Que (100 µm), Que‐FeMPF (100 µm), and Que‐CeMPF (100 µm), and incubated for 3 h at 37 °C. After incubation, the samples were centrifuged at 1000 g for 5 min, and the absorbance of the supernatant was measured at 545 nm using a microplate reader. RBCs exposed to 0.1% Triton X‐100 served as the negative control, and those exposed to PBS served as the positive control.

(5) $$\begin{array}{ccc} \mathrm{Hemolysis}\left(\%\right) & = & \left(OD_{\mathrm{MPF}}-OD_{\mathrm{Triton}\;x-100}\right)/\left(OD_{\mathrm{PBS}}-OD_{\mathrm{Triton}\;x-100}\right)\\ & & \times 100\% \end{array}$$

### Inflammation Modulation of Que‐Fe‐CeMPF In Vitro

Murine macrophage cells (RAW 264.7, ATCC TIB‐71) were obtained from the American Type Culture Collection and cultured in Dulbecco's modified Eagle's medium with high glucose containing 10% FBS and 1% penicillin/streptomycin (DMEM‐HG), at 37 °C in a humidified incubator with 5% CO_2_. The macrophages (5*10^5^ cells/well) were seeded in a 6‐well plate and cultured at 37 °C for 24 h. Cells were then stimulated with LPS (200 ng mL^−1^) in DMEM‐HG for 1 h, followed by treatment with 50 µm Que, Que‐FeMPF (equivalent concentration Que), Que‐CeMPF (equivalent concentration Que), Que‐Fe‐CeMPF (equivalent concentration Que) for another 12 h at 37 °C. The cell suspensions were centrifuged, and the amount released TNF‐α, IL‐6, and IL‐10 in the supernatants was determined with ELISA kits according to the manufacturer's protocol. Briefly, the supernatants were incubated with biotin‐labelled anti‐mouse TNF‐α (IL‐6 or IL‐10) antibody and formed an immune complex. Upon the addition of TMB, a blue color develops via horseradish peroxidase (HRP) catalysis, which turns yellow after adding the stop solution (2 m H_2_SO_4_). The OD value was measured at 450 nm using a microplate reader, and the cytokine concentrations were determined using standard curves.

### Evaluation of Que‐Fe‐CeMPF Against P. Aeruginosa in a Murine Model of Pulmonary Bacterial Infection

Female ICR mice (4–6 weeks, 22–24 g) were purchased from Zhejiang Vital River Laboratory Animal Technology Co., Ltd, and were housed under specific pathogen‐free conditions. The animal experimental protocol was approved by the Institutional Animal Care and Use Committee, Wenzhou Institute, University of Chinese Academy of Sciences (No. WIUCAS21071223), in accordance with the Administration Regulations governing the Affairs of Experimental Animals under the jurisdiction of the Ministry of Science and Technology in China.

Female ICR mice were used to establish a pulmonary infection model via intratracheal administration of *P. aeruginosa* Xen41 (20 µL, 10^6^ CFU/mL) in PBS under isoflurane anesthesia.^[^ ^51^ ^]^ After 1‐day post‐infection, the mice were randomly assigned into four groups of six animals each, and treated via intratracheal administration of 20 µL PBS (negative control), ciprofloxacin (positive control, 100 µg mL^−1^), 50 µm Que, or 50 µm Que‐Fe‐CeMPF (equivalent concentration of Que) with H_2_O_2_ (100 µm). The treatment was repeated on days 2 and 3. At day 4, 500 µL of blood was collected from three mice via orbital sampling into tubes containing anticoagulant EDTA for routine hematological analysis. The mice were sacrificed, and lung tissues from mice were collected and homogenized in 10 mL PBS using an ultrasonic cell homogenizer (JY92‐IIN, Shanghai, China). The supernatant was obtained by centrifugation of the collected tissues at 3000 g for 20 min at 4 °C and serially diluted, plated on LB agar plates. After incubation at 37 °C for 18 h, CFUs were counted. The cytokine levels in lung homogenates were determined as described above.

Simultaneously, major organs, including the heart, liver, spleen, lung, and kidney, were collected for histopathological analysis via hematoxylin and eosin (H&E) staining. Briefly, the organs were fixed in 10% formalin for 12 h, dehydrated with a series of ethanol solutions (70%, 80%, 90%, 95%, 100%) for 30 min each, embedded in paraffin wax, and sectioned into 4 µm thick slices and stained with H&E stain. The stained tissue sections were analyzed using a light microscope.

Immunofluorescence analysis was used to identify the different macrophage phenotypes within the lung tissue. This approach used specific markers: MPO (myeloperoxidase), F4/80 (monoclonal antibody), CD86 (monoclonal antibody), and CD206 (mannose receptor), allowing comprehensive characterization of macrophage subtypes. MPO and F4/80 are markers for macrophage activation and maturation, while CD86 is expressed by M1 macrophages and CD206 by M2 macrophages. Briefly, lung tissue was fixed in 4% paraformaldehyde to preserve cellular structures and embedded in paraffin for sectioning. Sections were incubated in 5% FBS for 1 h. Tissue sections were incubated overnight at 4 °C with primary antibodies specific to macrophages. The tissue sections were then washed multiple times with PBS to remove any unbound primary antibodies. Fluorescence‐labelled secondary antibodies (goat anti‐mouse IgG H&L, Alexa Fluor 488) were then applied for 1 h, which specifically recognize the primary antibodies. Finally, the stained tissue sections were analyzed using a fluorescence microscope equipped with appropriate filter sets for the fluorophores used.

### Statistical Analysis

All data were expressed as means ± SD values. A one‐way ANOVA was used to determine the statistical significance between groups. GraphPad Prism 8 and Microsoft Excel 2019 software were used for data processing and statistical analysis. A P‐value of < 0.05 was considered statistically significant.

## Conflict of Interest

The authors declare no conflict of interest.

## Author Contributions

Y.W., H.C.v.d.M., Y.F.L., Y.L., and L.Q.S. conceived and designed the experiments. Y.W. and F.W. conducted the synthesis and characterization of Que‐Fe‐CeMPF. Y.W., L.H., C.G., and S.W. assessed the biosafety and anti‐inflammation properties of Que‐Fe‐CeMPF. Y.W., F.W., and Y.F.L. evaluated the in vitro and in vivo antibacterial efficacy. Y.W., H.C.v.d.M., and Y.L. drafted the manuscript. H.C.v.d.M., Y.L., Y.R., Y.F.L., and L.Q.S. revised the manuscript. All authors contributed to data analysis and paper preparation.

## Supporting information



Supporting Information

## Data Availability

The data that support the findings of this study are available from the corresponding author upon reasonable request.
