# Supplementary material for: Bimetal‐Phenolic Framework to Combat Bacterial Infections via Synergistic Biofilm Dispersal, Bacterial Killing and Immune Modulation
Source: Adv Sci (Weinh). 2025 Sep 17;12(45):e13863. doi: 10.1002/advs.202513863 (PMC12677615; doi:10.1002/advs.202513863)
Supplement: Supplementary file 1 — Supporting Information [file ADVS-12-e13863-s001.docx]

**Supporting Information**

**Bimetal-Phenolic Framework to Combat Bacterial Infections via Synergistic Biofilm Dispersal, Bacterial Killing and Immune Modulation**

Yaran Wang^a,b,c^, Fan Wu^a,b,c^, Lei Hua^a^, Chang Gao^a,e^, Siran Wang^a,b,c^, Yong Liu^b,a,e,^*, Yijin Ren^d^, Linqi Shi^b,^*, Henny C. van der Mei^c,^*, and Yuanfeng Li^a^*

a. Consortium for Infection and Innovation (CII), Translational Medicine Laboratory, the First Affiliated Hospital of Wenzhou Medical University, Wenzhou, Zhejiang 325035, China

b. State Key Laboratory of Medicinal Chemical Biology, Key Laboratory of Functional Polymer Materials, Ministry of Education, Institute of Polymer Chemistry, College of Chemistry, Nankai University, Tianjin, 300071, China

c. University of Groningen and University Medical Center Groningen, Department of Biomaterials & Biomedical Technology, Groningen, 9713 AV, Netherlands

d. University of Groningen and University Medical Center Groningen, Department of Orthodontics, Groningen, 9700 RB, Netherlands

e. Wenzhou Institute, University of Chinese Academy of Sciences, Wenzhou, Zhejiang 325001, China

Corresponding authors: Yuanfeng Li (yuanfengli@wmu.edu.cn), Henny C. van der Mei (h.c.van.der.mei@umcg.nl), Linqi Shi ([shilinqi@nankai.edu.cn](mailto:shilinqi@nankai.edu.cn)) and Yong Liu ([y.liu@nankai.edu.cn](mailto:y.liu@nankai.edu.cn))


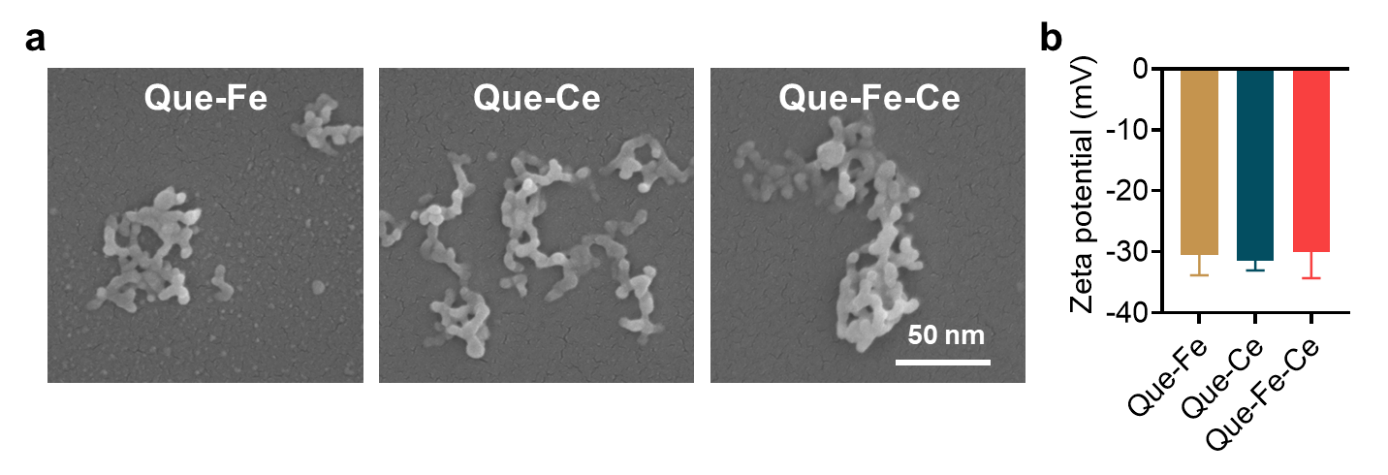


**Figure S1.** (**a**) SEM images and (**b**) Zeta potentials of Que-Fe, Que-Ce, and Que-Fe-Ce MPFs in PBS. Error bars represent the standard deviation of triplicate experiment.


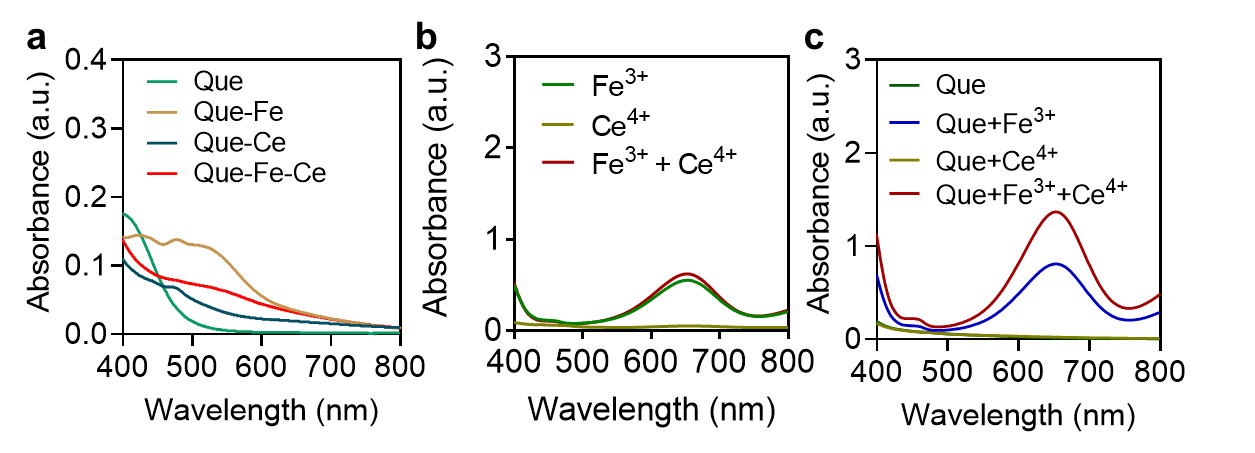


**Figure S2.** UV−vis absorption spectra of TMB oxidation under various conditions.

(**a**) Absorbance spectra of TMB oxidation of 50 µM Que, Que-Fe, Que-Ce and Que-Fe-Ce after 10 min at pH 4.5 in the absence of H_2_O_2_.

(**b**) Absorbance spectra of TMB oxidation of 50 µM Fe^3+^ ions, Ce^4+^ ions and a direct mixture of Fe^3+^ and Ce^4+^ ions after 10 min at pH 4.5 in the presence of H_2_O_2_ (100 μM).

(**c**) Absorbance spectra of TMB oxidation of 50 µM Que alone and after addition of Que to metal ions and directly mixed for 10 min at pH 4.5 in the presence of H_2_O_2_ (100 μM).

**Figure S3.** The Lineweaver-Burk activity plot of 50 µM Que-Fe-Ce for different concentration of H_2_O_2_.


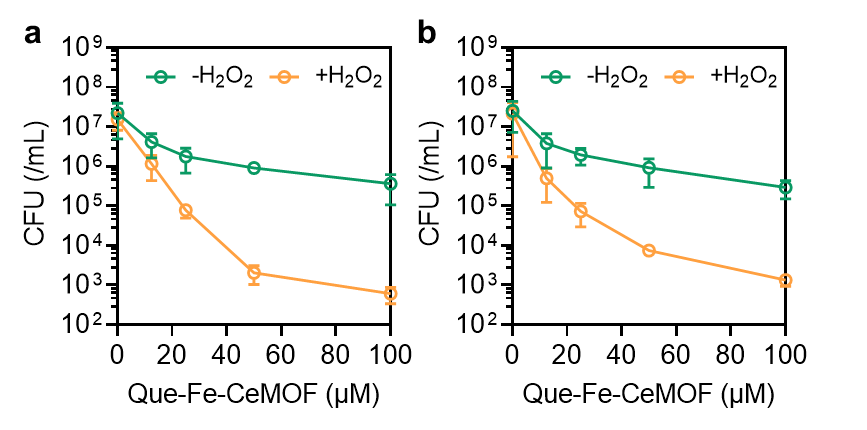


**Figure S4.** Antibacterial activity of Que-Fe-CeMPF against planktonic bacteria as a function of the concentration of Que-Fe-CeMPF in the presence and absence of H_2_O_2_ (100 μM).

(**a**) *S. aureus* Xen36 and (**b**) *P. aeruginosa* Xen41 (10^7^/mL) exposed to Que-Fe-CeMPF at different concentrations (0, 12.5, 25, 50 and 100 μM) for 3 h in sodium acetate buffer at pH 4.5. Error bars represent the standard deviation of triplicate experiments.


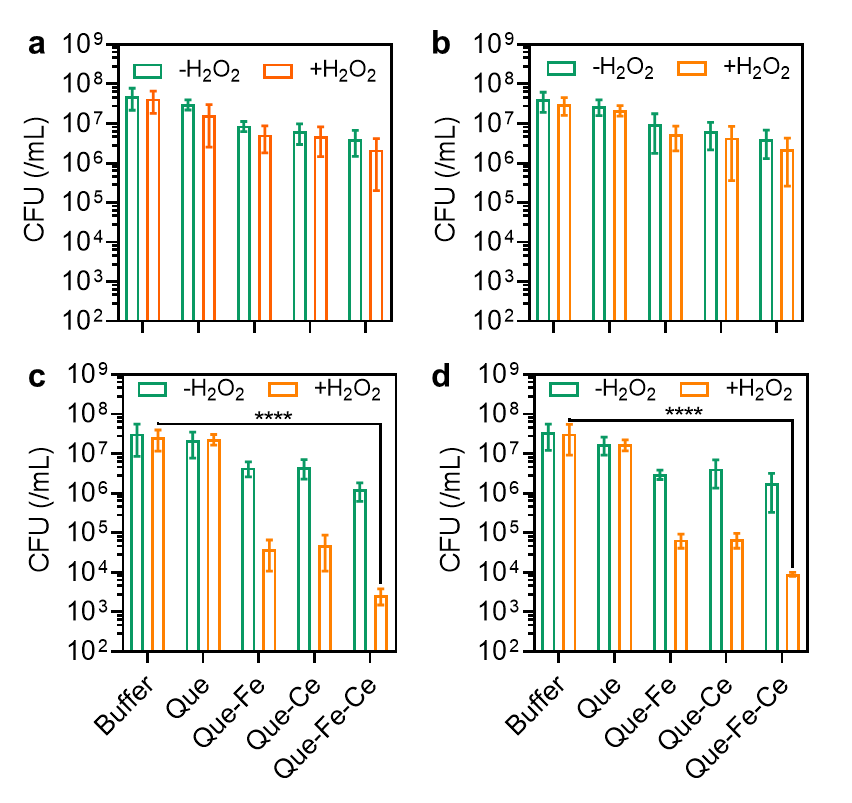


**Figure S5.** Antibacterial activity of Que and different MPFs against planktonic bacteria in the presence and absence of H_2_O_2_ (100 μM).

(**a**) *S. aureus* Xen36 and (**b**) *P.* *aeruginosa* Xen41 (10^7^/mL) exposed to 50 μM of Que, Que-FeMPF, Que-CeMPF, or Que-Fe-CeMPF (all containing equivalent concentrations of Que) for 3 h in sodium acetate buffer at pH 7.4. (**c**) Same as panel **a**, but now at pH 4.5. (**d**) Same as panel **b**, but now at pH 4.5. ****p < 0.0001 indicate statistical significance (one-way ANOVA) over the differences indicated by the spanning bars. Error bars represent the standard deviation of triplicate experiments.


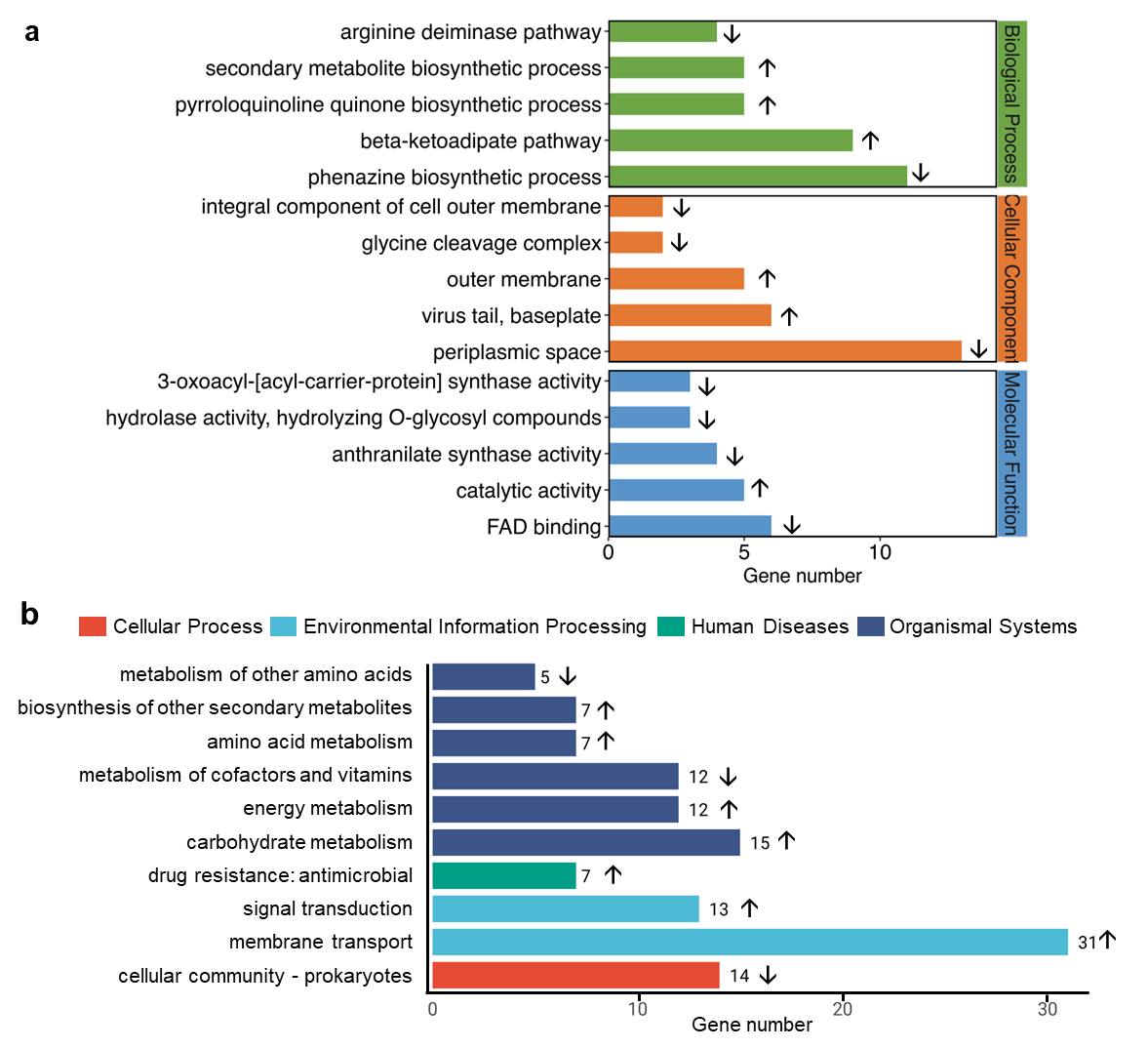


**Figure S6.** Transcriptomic analysis of *P. aeruginosa* exposed to ciprofloxacin. (**a**) GO pathway analysis and (**b**) KEGG pathway analysis of diﬀerentially expressed genes in *P. aeruginosa* exposed to ciprofloxacin, compared to PBS-treated controls. ↑ indicates upregulation; ↓ indicates downregulation.


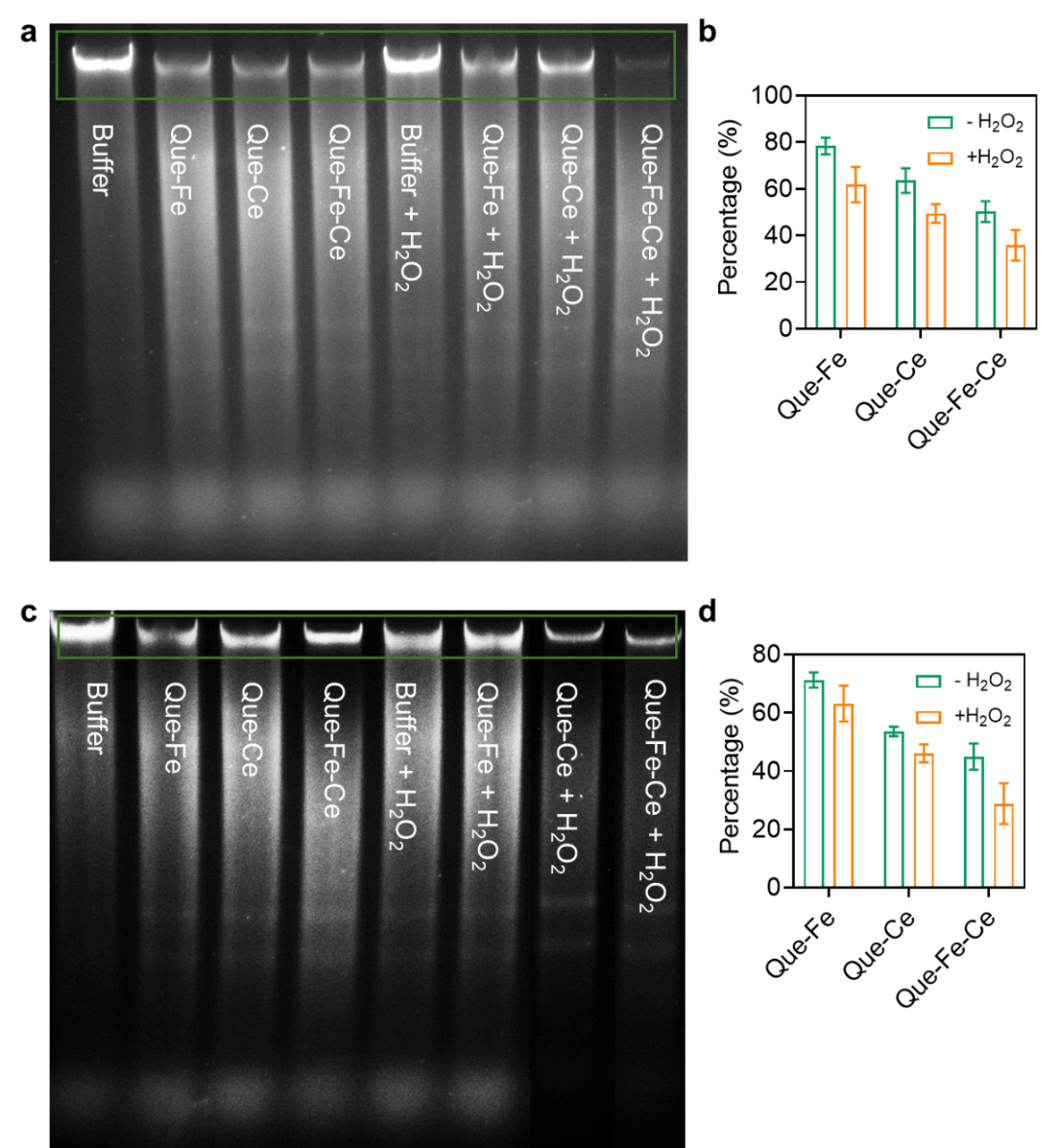


**Figure S7.** Degradation of the biofilm matrix of *S. aureus* Xen36 and *P. aeruginosa* Xen41.

(**a**) Agarose gel electrophoresis of degraded *S. aureus* Xen36 biofilm after exposure to 50 μM MPFs for 3 h. (**b**) Corresponding gray value quantification from panel a, expressed as a percentage relative to *S. aureus* Xen36. (**c**) Same as panel **a**, but for *P. aeruginosa* Xen41. (**d**) Same as panel **b**, but for *P. aeruginosa* Xen41. Percentage (100%) are relative to biofilm matrix exposed to buffer at pH 4.5 in the absence or presence of H_2_O_2_ (100 µM).


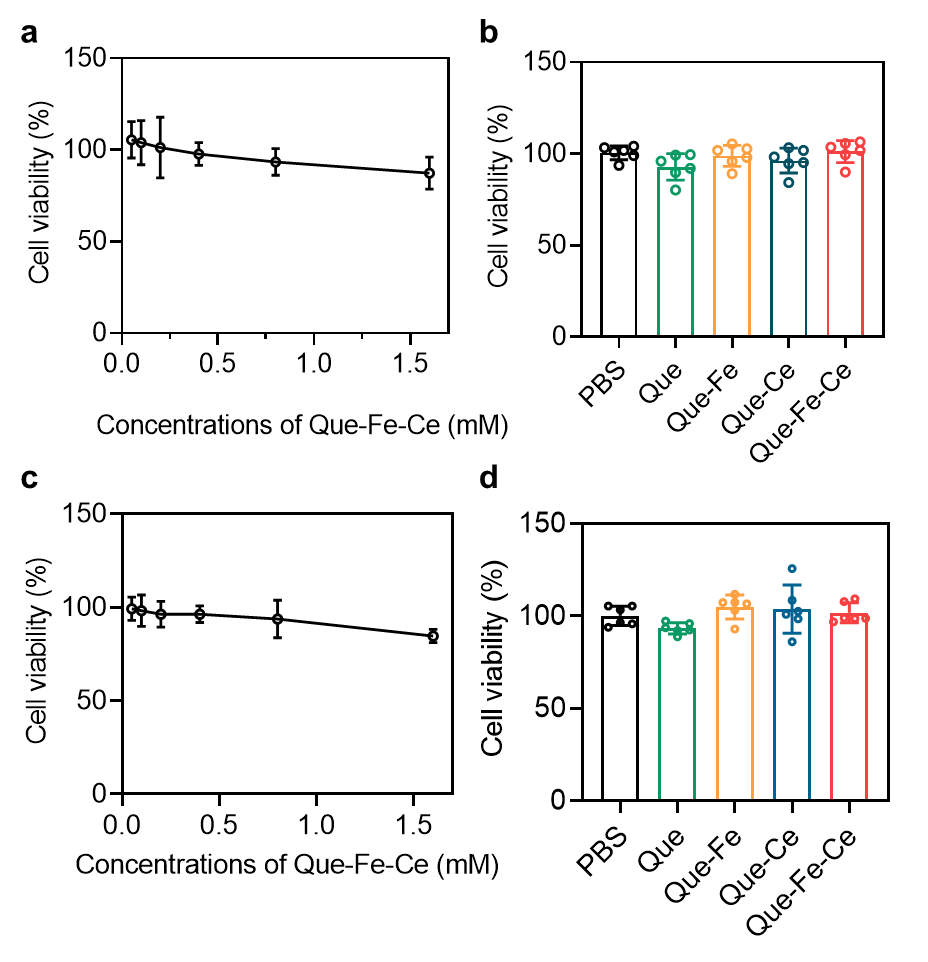


**Figure S8.** Cytotoxicity of Que and different MPFs with equivalent concentrations of Que towards fibroblasts and human umbilical vein endothelial cells (HUVEC).

(**a**) Viability of fibroblasts exposed for 24 h as a function of Que-Fe-CeMPF concentration, and (**b**) Viability of fibroblasts exposed to Que (50 µM) and different MPFs (50 µM, equivalent concentrations of Que). (**c**) Same as panel **a**, but for HUVEC in the appropriate growth medium. (**d**) Same as panel **b** but HUVEC in the appropriate growth medium. Cell viability was determined by measuring metabolic activity and is expressed relative to untreated cells cultured in the appropriate growth medium.


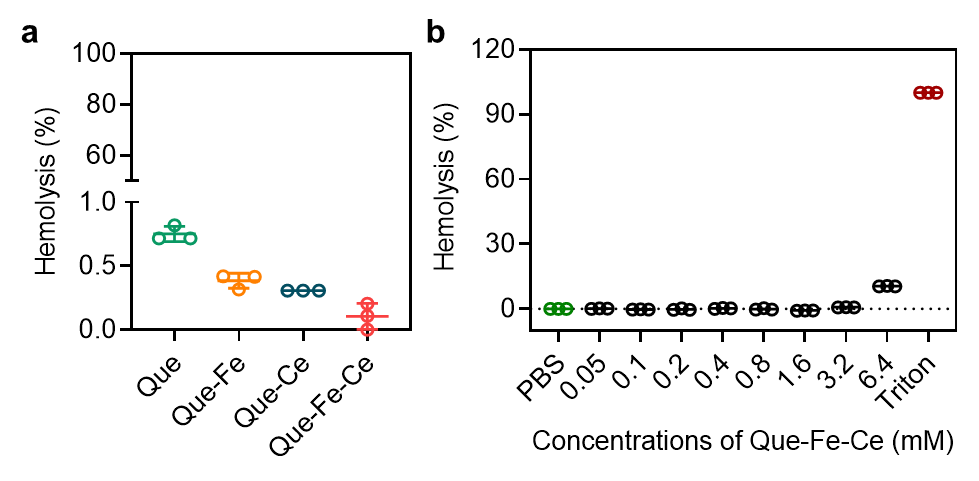


**Figure S9.** Hemolysis of red blood cells (RBCs) from mice after exposure to Que and different MPFs.

(**a**) RBCs exposed for 3 h to 50 μM Que and 50 μM of the different MPFs (equivalent concentration of Que) in the presence of H_2_O_2_ (100 µM). (**b**) RBCs exposed to different concentrations Que-Fe-CeMPF in the presence of H_2_O_2_ (100 µM). Hemolysis was quantified relative to PBS (0%, negative control) and 0.1% Triton X-100 (100%, positive control).


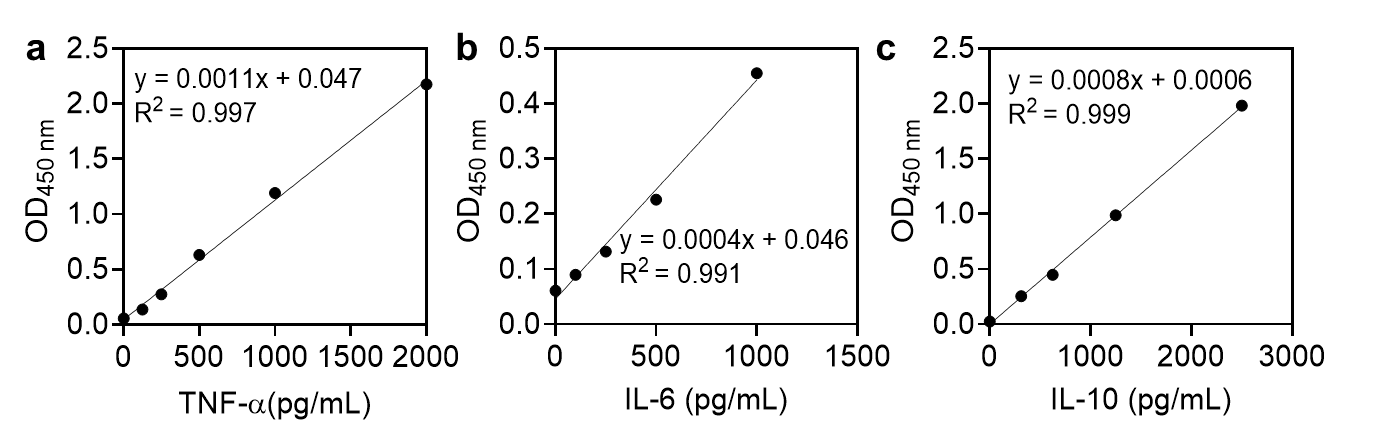


**Figure S10.** Standard curves for TNF-α, IL-6, and IL-10 cytokines quantified using ELISA kits. The absorbance at 450 nm was plotted as a function of the cytokine concentration.


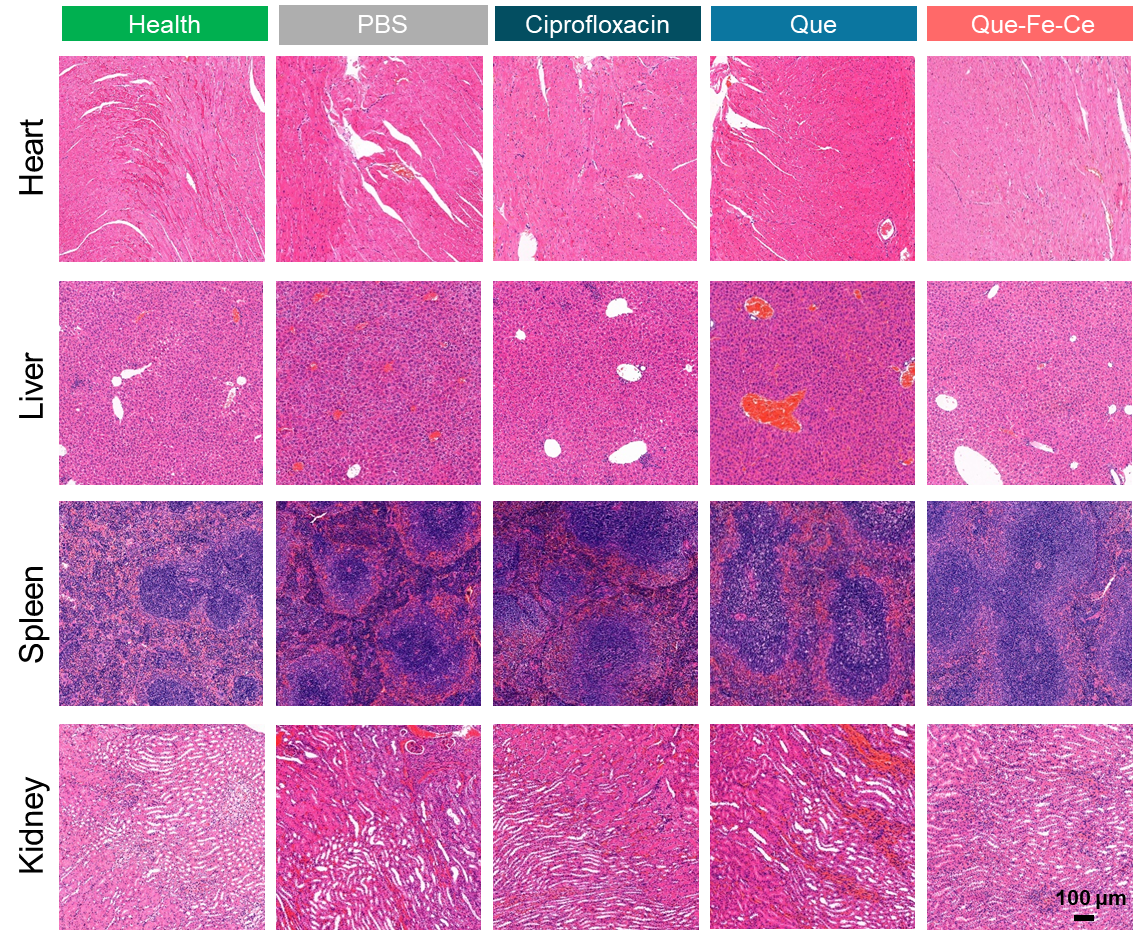


**Figure S11.** Histologic slices of heart, liver, spleen, and kidney from healthy mice or pneumonia-infected mice after intratracheal administration of 20 μL PBS, ciprofloxacin (100 μg/mL), 50 μM Que and 50 μM Que-Fe-CeMPF in the presence of H_2_O_2_ (100 µM). Mice were treated over three consecutive days.

**Figure S12.** Blood parameters with healthy ranges indicated by dotted lines in healthy mice and pneumonia-infected mice after treatment with 20 μL PBS, ciprofloxacin (100 μg/mL), 50 μM Que and 50 μM Que-Fe-CeMPF in the presence of H_2_O_2_ (100 µM) over three consecutive days. WBC: white blood cell; RBC: red blood cell; HGB: hemoglobin; PLT: platelet count; HCT: hematocrit; MCH: mean corpuscular hemoglobin; MCV: mean corpuscular volume; MCHC: mean corpuscular hemoglobin concentration. Error bars represents data from three mice.
